# Direct interaction between MreB and the RodA‐PBP2 complex organizes lateral cell wall synthesis in *Escherichia coli*

**DOI:** 10.1002/mlf2.70079

**Published:** 2026-05-18

**Authors:** Rui Zhan, Han Gong, Ying Li, Xiangdong Chen, Joe Lutkenhaus, Shishen Du

**Affiliations:** ^1^ State Key Laboratory of Metabolism and Regulation in Complex Organisms College of Life Sciences, Wuhan University Wuhan China; ^2^ Hubei Key Laboratory of Cell Homeostasis College of Life Sciences, Wuhan University Wuhan China; ^3^ State Key Laboratory of Virology and Biosafety College of Life Sciences, Wuhan University Wuhan China; ^4^ Department of Microbiology, Molecular Genetics and Immunology University of Kansas Medical Center Kansas City Kansas USA

**Keywords:** bacterial cell wall, elongasome, MreB, peptidoglycan, RodA‐PBP2

## Abstract

The elongasome, or the Rod complex, orchestrates lateral peptidoglycan (PG) synthesis in many rod‐shaped bacteria. It consists of the actin‐like protein MreB, the PG synthase RodA‐PBP2 complex, as well as MreCD and RodZ. Although the loss or disruption of any elongasome component results in a loss of rod shape, previous studies found that a constitutively active RodA‐PBP2 complex can partially bypass the requirement of MreCD and RodZ for lateral PG synthesis and restore rod shape. However, how MreB is connected to RodA‐PBP2 under this situation and whether this linkage is important for elongasome activity in wild‐type cells remain unknown. Here, we report the isolation of additional RodA and PBP2 variants that can partially compensate for the absence of MreCD and RodZ in lateral PG synthesis. Taking advantage of these mutants and guided by an AlphaFold 3 structural model of the elongasome complex, we discover that both the cytoplasmic region of PBP2 and the C‐terminal tail of RodA interact with MreB. Moreover, disruption of these interactions results in a loss of rod shape, indicating that the interaction between MreB and RodA‐PBP2 is critical for elongasome function. Taken together, our results uncover how the MreB cytoskeleton is coupled to RodA‐PBP2 to organize lateral PG synthesis. These findings provide mechanistic insights into cell wall biogenesis in bacteria and offer strategies for the development of new antibiotics targeting the elongasome.

## Impact statement

Assembly of the lateral peptidoglycan (PG) cell wall in many rod‐shaped bacteria is carried out by the elongasome, or the Rod complex, consisting of the cytoskeletal protein MreB, the PG synthase RodA‐PBP2, and three regulatory proteins MreC, MreD, and RodZ. However, how these proteins interact with each other to orchestrate lateral PG synthesis remains incompletely understood. Here, we discover that MreB interacts directly with both RodA and PBP2 to organize lateral PG synthesis. These findings reveal the coupling mechanism of cytoskeletal protein and PG synthase, and may aid the development of inhibitors of cell wall synthesis.

## INTRODUCTION

Bacteria show remarkable diversity in shape and size. The peptidoglycan (PG) mesh layer, which is a crucial component of the bacterial cell wall, determines the morphology and size of bacterial cells^1^, ^2^, ^3^. This layer not only provides structural support for bacterial cells but also protects them from osmotic pressure. PG is composed of long glycan strands containing repeating disaccharide units of *N*‐acetylmuramic acid (MurNAc) and *N*‐acetylglucosamine (GlcNAc) linked by β‐1,4‐glycosidic bonds. Adjacent glycan strands are crosslinked to each other via peptide bridges^3^. The PG precursor lipid II, a disaccharide pentapeptide, is synthesized within the cell and flipped to the outer leaflet of the cell membrane, where it is polymerized into glycan chains by PG glycosyltransferases (PGTase). Nascent glycan strands are subsequently crosslinked into the existing PG network by transpeptidases (TPase)^1^, ^4^, ^5^. There are two types of PG synthases: one is the class A penicillin binding protein (aPBP) containing both PGTase and TPase activities, and the other consists of a complex formed by a SEDS (Shape, Elongation, Division, and Sporulation) family protein with PGTase activity and a cognate class B penicillin binding protein (bPBP) with TPase activity^1^. Most cells have two SEDS‐bPBP complexes, one synthesizes PG during cell elongation and another during cell division. The major role of aPBPs is believed to fortify the PG network to maintain its integrity^6^, ^7^, ^8^, ^9^, ^10^.

New PG material is not inserted into existing PG mesh randomly around the cells, but at specific locations to ensure the expansion and division of this matrix in the right place at the right time. Most rod‐shaped bacteria synthesize lateral PG and maintain their morphology through a muti‐protein complex called the elongasome, or the Rod complex, while synthesis of septal PG (sPG) at the division site is mediated by the divisome, a large protein complex consisting of dozens of components. The elongasome contains six highly conserved components, including the cytoskeletal protein MreB, the PG synthase RodA‐PBP2 (a SEDS‐bPBP complex), and the regulatory proteins MreCD and RodZ^1^, ^2^. In *Escherichia coli*, the absence or inactivation of any elongasome component results in cell growth defects and the formation of spherical cells^11^, ^12^, ^13^, ^14^, ^15^, ^16^. Moreover, antibiotics targeting the elongasome, such as the β‐lactam mecillinam, which inhibits PBP2 activity^17^, have been widely used in clinical settings, underscoring the importance of the elongasome for bacterial cell shape maintenance and survival.

Although the elongasome has been extensively studied, not all functions of its components and their interrelationships are fully understood. MreB, a homolog of eukaryotic actin, binds to ATP and forms anti‐parallel double filaments with an intrinsic curvature on the inner leaflet of the cytoplasmic membrane^12^, ^18^, ^19^. Inactivation of MreB by mutation or treatment with its inhibitor A22, which prevents MreB assembly^18^, ^20^, ^21^, ^22^, results in shape defects and cell growth arrest, indicating a crucial role for MreB in cell shape maintenance and growth. Interestingly, MreB filaments have been shown to rotate perpendicularly to the cell's long axis, driven by active lateral PG synthesis by RodA‐PBP2 but not its ATPase activity^23^, ^24^, ^25^, ^26^. Moreover, MreB interacts with components of the elongasome, including MreC and RodZ^22^, ^27^. A co‐crystal structure of MreB and RodZ from *Thermotoga maritima* shows that the cytoplasmic helix–turn–helix (HTH) domain of RodZ directly interacts with MreB^18^, ^28^, ^29^, ^30^. Deletion of *rodZ* or disruption of the RodZ‐MreB interaction leads to the formation of aberrant MreB structures and cell shape defects, suggesting that RodZ modulates the assembly of MreB filaments. Currently, MreB filaments are believed to function as rudders for lateral PG synthesis in rod‐shaped bacteria^1^, ^31^.

RodA is a multi‐transmembrane protein belonging to the widely conserved SEDS family of PG polymerases^6^, ^32^. It forms a tight complex with PBP2, which has a small N‐terminal cytoplasmic domain, a transmembrane (TM) domain, and a large C‐terminal periplasmic domain with TPase activity^9^, ^32^. The structures of the RodA‐PBP2 complex from *Thermus thermophilus* and *E. coli* revealed that RodA and PBP2 form a complex in a 1:1 stoichiometry with two interaction interfaces: one in the membrane plane via interactions between the TM domains and the other in the periplasm between the hinge region and the pedestal domain of PBP2 and the extracellular loop 4 (ECL4) of RodA^9^, ^33^. Mutations within the periplasmic interaction interface of the RodA‐PBP2 complex can result in enhancement or inhibition of lateral PG synthesis, indicating a crucial role of this interaction interface in controlling the activity of the complex^9^, ^34^, ^35^.

Although purified RodA‐PBP2 complexes from a number of bacteria display PGTase activity *in vitro*, accumulating evidence indicates that the complex is stimulated *in vivo* ^27^, ^36^, ^37^, ^38^. Dominant‐negative mutations in MreC (such as G156D and R292H), which block lateral PG synthesis and result in cell growth and shape defects, have been reported^35^, ^38^. Interestingly, mutations in RodA (A234T) or PBP2 (L61R) can suppress the defects caused by these MreC mutations^35^, ^38^. *In vivo* and *in vitro* characterization of the RodA‐PBP2^L61R^ complex showed that it displayed increased PGTase activity in comparison to wild‐type RodA‐PBP2, suggesting that PBP2^L61R^ is an activating mutation of the complex and the deficiency of the MreC variants is an inability to stimulate the activity of RodA‐PBP2. A structure of the MreC‐PBP2 complex from *Helicobacter pylori* reveals that MreC's periplasmic domain interacts with the pedestal domain of PBP2 and induces a conformational change in PBP2^39^. Thus, it has been proposed that the binding of MreC to PBP2 allosterically activates the PGTase activity of the RodA‐PBP2 complex^35^, ^40^. In addition to MreCD, RodZ is likely also necessary for the full activity of the elongasome *in vivo*. Deletion of *rodZ* results in aberrant assembly of MreB and the formation of spherical cells. Activating RodA‐PBP2 variants can restore rod shape to Δ*rodZ* cells^35^, ^41^, indicating that increasing RodA‐PBP2 activity compensates for the absence of RodZ and may restore proper MreB localization. Strikingly, PBP2^L61R^ can support the growth of an *mreCD* and *rodZ* triple‐deletion strain and partially restore rod shape^35^, implying that MreB is directly connected to the RodA‐PBP2 complex, but how this occurs has not been determined.

In this study, we characterize additional mutations in RodA and PBP2 that confer resistance to A22 and rescue the growth and shape of an *mreCD* and *rodZ* triple‐depletion strain in *E. coli*. Using a combination of genetic, biochemical, and cytological approaches, we show that MreB interacts with both PBP2 and RodA, and these interactions are important for elongasome activity. These findings unravel the coupling mechanism between MreB and the RodA‐PBP2 complex, deepen our understanding of the elongasome, and provide clues for designing its inhibitors.

## RESULTS

### MreB and a RodA‐PBP2 mutant complex can form a minimal elongasome

A previous study showed that PBP2^L61R^, which results in a constitutively active RodA‐PBP2 complex, provides resistance to the MreB antagonist A22 and partially rescues the growth and shape defects of the Δ*mreCD*Δ*rodZ* strain^35^. Characterization of this mutant revealed a central role of PBP2 in activating the PGTase activity of RodA. Isolation and investigation of similar mutants would likely provide novel insights into the mechanisms governing the assembly and activity of the elongasome. Therefore, we attempted to isolate additional RodA and PBP2 mutants that displayed phenotypes similar to PBP2^L61R^, such as those (i) provide resistance to A22; (ii) rescue growth and rod shape defects of Δ*mreCD*, Δ*rodZ,* or Δ*mreCD*Δ*rodZ* cells; and (iii) suppress the growth and shape defects caused by inactivating mutations in elongasome components.

**Figure 1:**
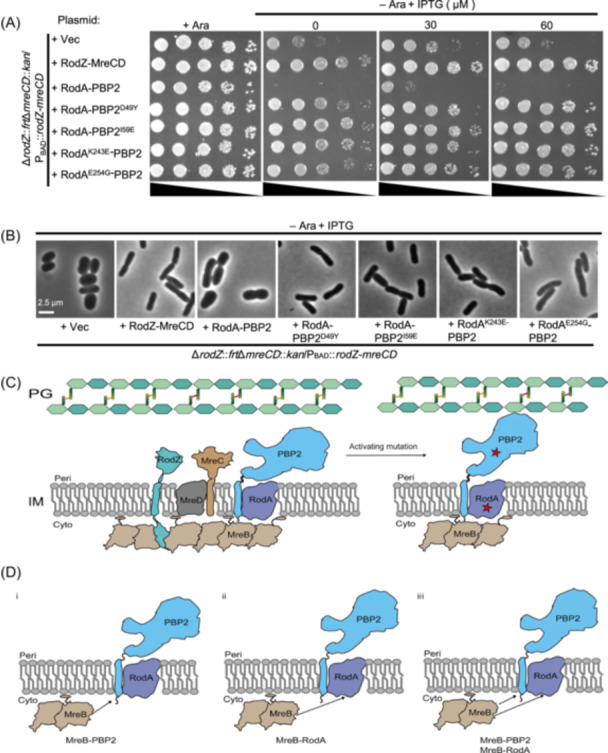
MreB and a mutant RodA‐PBP2 complex form a minimal elongasome, indicating a direct association between them. (A) RodA‐PBP2 mutants rescued growth of the RodZ and MreCD depletion strain. Plasmid pDSW210, pZR187 (pDSW210, P_206_::*rodZ‐mreCD*), pSD314 (pDSW210, P_206_::*pbp2‐rodA*), or its derivatives carrying different alleles of *pbp2‐rodA* were transformed into strain RZ96A (TB28, Δ*rodZ*::*frt*Δ*mreCD*::*kan*/pZR186(P_BAD_::*rodZ‐mreCD*)) on LB plates with antibiotics and 0.2% arabinose at 37°C. The next day, a single transformant of the resulting strains was resuspended in 1 ml of LB medium and serially diluted in 10‐fold. 2.5 μl of each dilution was spotted on LB plates with antibiotics and the indicated concentration of IPTG. Plates were incubated at 37°C overnight and photographed. (B) RodA or PBP2 variants suppressed the shape defects of RodZ‐ and MreCD‐depleted cells. Scale bar, 2.5 μm. Overnight cultures of the strains from (A) were diluted 1:100 in fresh LB medium with antibiotics and 0.2% arabinose, and grown at 37°C for 2 h. Cells were then collected by centrifugation and washed twice with fresh LB and resuspended in the same volume of LB medium. These arabinose‐free cultures were diluted 1:100 in fresh LB medium with antibiotics and grown at 37°C for 2–3 h. Then, cultures were diluted 1:10 in 5 ml of fresh LB medium again and IPTG was added to a final concentration of 30 μM and grown at 37°C for 2–3 h. Cells were immobilized on a 2% agarose pad for photography. (C) Schematic depicting the effects of various mutations in *pbp2* or *rodA* on the function of the elongasome. Left: *E. coli* elongasome consists of MreB, MreC, MreD, the RodA‐PBP2 complex, and RodZ, all of which are necessary for lateral PG synthesis in wild‐type cells. Right: MreB and mutant RodA‐PBP2 complexes form a minimal elongasome, bypassing the need for MreCD and RodZ for lateral PG synthesis. (D) Possible scenarios for the association between MreB and RodA‐PBP2: (i) MreB only interacts with PBP2; (ii) MreB only interacts with RodA; and (iii) MreB interacts with both PBP2 and RodA. Ara, arabinose; Cyto, cytoplasmic; IM, inner membrane; Peri, periplasmic; PG, peptidoglycan.

L61 is located in the hinge region between the TM and the large periplasmic domain of PBP2, which interacts extensively with ECL4 of RodA (Figure S1)^9^. This interaction interface between RodA and PBP2 is believed to play an important role in regulating RodA‐PBP2 activity. Therefore, we substituted residues in this interaction interface with alanine or negatively charged amino acids using site‐directed mutagenesis based on the *E. coli* RodA‐PBP2 structure^33^. The mutants were expressed from an IPTG‐inducible promoter in a plasmid and tested for complementation and resistance to A22 in a RodA or RodA‐PBP2 depletion strain. In these strains, wild‐type RodA or RodA‐PBP2 was expressed from a compatible plasmid under an arabinose‐inducible promoter. In the absence of arabinose, these two strains would not be able to grow. We successfully isolated A22‐resistant mutations in the hinge region of PBP2 and the ECL4 of RodA, as well as loss‐of‐function mutations resulting in PBP2 or RodA variants that could not rescue cell growth (Figures S1 and S2). Similar to PBP2^L61R^, several of the A22‐resistant PBP2 or RodA mutations, when introduced into the chromosome, partially rescued the growth and shape defects of Δ*rodZ* cells (Figure S3A‐B). Moreover, ectopic expression of these mutants (PBP2^D49Y^, PBP2^I59E^, RodA^K243E^, and RodA^E254G^) also partially rescued the growth and morphology defects of RodZ‐MreCD‐depleted cells (Figure 1A,B), in which wild‐type RodZ‐MreCD supplied from a plasmid under the control of an arabinose‐inducible promoter could be depleted by removing arabinose. These mutations also suppressed the growth and shape defects caused by dominant‐negative MreC mutations (this will be reported in preparation). Thus, these mutations increase the activity and/or integrity of the elongasome, resembling the activating mutation PBP2^L61R^. The phenotypes of these mutants, along with the previous findings with PBP2^L61R^, indicate that MreB and the RodA‐PBP2 complex constitute a minimal elongasome in the presence of mutations enhancing elongasome function (Figure 1C). This also strengthens the notion that MreB contacts RodA‐PBP2 directly.

There are three possible ways for MreB to associate with RodA‐PBP2 to form the minimal elongasome: (i) MreB interacts only with PBP2; (ii) MreB interacts only with RodA; and (iii) MreB interacts with both PBP2 and RodA (Figure 1D). To facilitate the determination of the interaction sites between MreB and the RodA‐PBP2 complex, AlphaFold 3 (AF3) was used to generate a structural model of the entire elongasome complex (RodZ‐MreB‐MreC‐MreD‐RodA‐PBP2) using a 1:1:1:1:1:1 stoichiometry. Although the model was not of high confidence, it predicted the interaction interface between RodA and PBP2, the interaction interface between MreB and RodZ, and that between MreC and PBP2 (Figure S4), suggesting a certain degree of accuracy. Interestingly, the model also indicated that the N‐terminal cytoplasmic domain of PBP2 and the cytoplasmic loops and the C‐terminal tail of RodA contact MreB (Figure S4). This structural model, along with the above mutations in RodA and PBP2, enabled us to probe the interaction between MreB and the RodA‐PBP2 complex, and its importance for lateral PG synthesis.

### PBP2 interacts directly with MreB and their interaction is important for lateral PG synthesis

**Figure 2:**
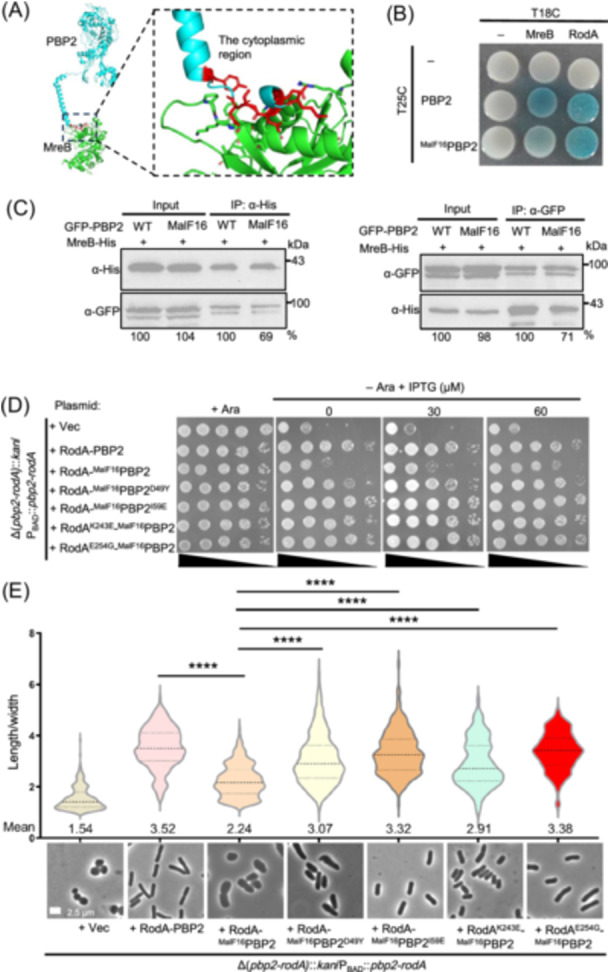
PBP2 interacts with MreB directly via its N‐terminal cytoplasmic domain. (A) A structural model of the *E. coli* MreB‐PBP2 complex obtained with AlphaFold3 (AF3). The model was extracted from a structural model of the complete elongasome complex generated by AF3 (Figure S4), which suggests that the cytoplasmic domain of PBP2 interacts with MreB. Residues on PBP2 predicted to be involved in the PBP2‐MreB interaction are shown in stick form and colored red. (B) Examination of the interaction between MreB and PBP2 or its variants by a bacterial two‐hybrid (BTH) assay. Pairs of plasmids harboring *mreB′‐t18C‐mreB′* and *t25c‐pbp2* or its variants were co‐transformed into BTH101. Single transformants were then spotted on LB plates containing appropriate antibiotics, 100 μM IPTG, and 40 μg/ml X‐gal. The plates were incubated at 30°C overnight before photography. (C) Co‐immunoprecipitation analysis of the interaction between PBP2 and MreB. Overnight cultures of TB28 carrying the plasmid pZR289 (P_206_::*gfp‐pbp2/mreB‐his*) or pZR289‐^MalF16^PBP2 (P_206_::*gfp‐* ^*malF16*^ *pbp2/mreB‐his*) were diluted 1:100 in 50 ml of fresh LB medium with antibiotics and 60 μM IPTG, and grown at 37°C for 3 h. Cells were collected and lysed by sonication. Supernatants were incubated with antibody‐coated magnetic beads. Immunocomplexes were collected, resuspended in SDS‐PAGE sample buffer, and boiled for 10 min before they were loaded on the SDS‐PAGE gel for Western blot analysis. The intensity of each band was quantified by ImageJ. Data are representative of two independent biological replicates. (D, E) RodA‐PBP2 variants suppressed the growth (D) and shape (E) defects caused by ^MalF16^PBP2. Plasmid pDSW210, pSD314 (pDSW210, P_206_::*pbp2‐rodA*), pSD314‐MalF16(pDSW210, P_206_::^*malF16*^ *pbp2‐rodA*), or its derivatives were transformed into strain RZ50 and selected on LB plates with ampicillin, chloramphenicol, and 0.2% arabinose at 37°C. The spot test was performed as in Figure 1A. To examine cell morphology (E), overnight cultures of the strains from (D) were diluted 1:100 in LB medium with antibiotics and grown at 37°C for 2 h, and the sample preparation was carried out as described in Figure 1B. The aspect ratio of cells was analyzed using Image J. The ratio calculated for each cell was plotted as a violin plot, showing the 25th and 75th percentiles, with the mean at the center. Data are shown as the mean ± SD. Number of cells analyzed is more than 200. Statistical significance was determined using an unpaired *t* test with Welch's correction. ****p < 0.0001. Scale bar, 2.5 µm. SDS‐PAGE, sodium dodecyl sulphate polyacrylamide gel electrophoresis.

Based on the AF3 model of the elongasome complex, the N‐terminal cytoplasmic domain of PBP2 interacts with MreB (Figure 2A). To test this model, we first used the bacterial two‐hybrid (BTH) assay to determine if PBP2 interacts with MreB. As shown in Figure 2B, PBP2 displayed a strong interaction signal with MreB and RodA, in agreement with a previous study^27^. To test if the N‐terminal cytoplasmic domain of PBP2 was critical for this interaction, we constructed a PBP2 variant (^MalF16^PBP2), in which this region of PBP2 was replaced with the N‐terminal cytoplasmic tail of MalF (MalF^16^), a multipass TM protein. As expected, the interaction signal between ^MalF16^PBP2 and MreB was substantially weaker than that between PBP2 and MreB; however, its interaction with RodA was not affected (Figure 2B). Thus, the cytoplasmic domain of PBP2 seems to mediate its interaction with MreB but is not important for its interaction with RodA.

To confirm the interaction between PBP2 and MreB, we performed co‐immunoprecipitation (co‐IP) experiments in wild‐type cells expressing both GFP‐tagged PBP2 (GFP‐PBP2) and 6× His‐tagged MreB (MreB‐His). A protein band corresponding to MreB‐His was detected in immunocomplexes isolated with the anti‐GFP antibody in cells expressing both proteins, but not in cells expressing only one of the fusion proteins (Figure S5A). In reciprocal tests, a protein band corresponding to GFP‐PBP2 could be detected in immunocomplexes isolated with the anti‐His antibody (Figure S5A). The amount of MreB‐His or GFP‐^MalF16^PBP2 was substantially reduced in the immunocomplexes isolated from cells expressing this PBP2 variant (Figure 2C), suggesting that the N‐terminal domain of PBP2 was important for the interaction. Western blotting showed that GFP‐^MalF16^PBP2 was as stable as GFP‐PBP2 (Figure S5B), suggesting that the reduced level of proteins was due to impaired interaction instead of instability of GFP‐^MalF16^PBP2. To test if the remaining protein found in the immunocomplexes was due to an indirect association with the other elongasome components, we repeated the experiment in a RodZ‐MreCD depletion strain. The amount of MreB‐His in the immunocomplex isolated with the anti‐GFP antibody from RodZ‐MreCD‐depleted cells expressing both GFP‐^MalF16^PBP2 and MreB‐His was further decreased to about 50%, but it was not eliminated (Figure S5C). This was likely due to an interaction between MreB‐His and RodA, which forms a tight complex with GFP‐^MalF16^PBP2 and mediates an indirect association of MreB‐His with GFP‐^MalF16^PBP2 in the co‐IP experiment.

To test if the PBP2‐MreB interaction is important for elongasome function, ^MalF16^PBP2 was expressed in tandem with RodA from a plasmid (^*malF16*^ *pbp2‐rodA*) under an IPTG‐inducible promoter and tested for its ability to complement a RodA‐PBP2 depletion strain RZ50. RodA‐PBP2 is supplied from a plasmid under the control of an arabinose‐inducible promoter in strain RZ50, so that its growth depends on the presence of arabinose. As shown in Figure 2D,E, while the expression of RodA‐PBP2 from the complementing plasmid rescued the growth and shape defects of RZ50 cells in the absence of arabinose, RodA‐^MalF16^PBP2 failed to do so, indicating that the interaction between PBP2 and MreB is important for elongasome function in wild‐type cells.

We also substituted the putative interacting residues in MreB, as predicted by AF3, to alanine and tested if they affected MreB function and interaction with PBP2 (Figure S6A). Complementation tests showed that while most of the MreB mutants expressed in tandem with MreCD could complement an MreBCD depletion strain under the non‐permissive condition, MreB^K89A^CD failed to do so (Figure S6B). Microscopic analysis of the cells showed that MreBCD‐depleted cells expressing MreB^K89A^CD were spherical (Figure S6C), suggesting that this residue is critical for MreB function. Western blotting showed that MreB^K89A^ was expressed at similar levels as the wild‐type MreB when labeled with a FLAG tag, suggesting that the loss of function was not due to protein instability (Figure S6D). Moreover, BTH assays showed that the K89A mutation weakened the interaction between MreB and PBP2, but not the interaction between MreB and RodZ, indicating that this residue is critical for MreB's interaction with PBP2. Thus, mutations in either PBP2 or MreB can disrupt their interaction.

### The PBP2‐MreB interaction is important for lateral PG synthesis by a minimal elongasome

Since the above results showed that disruption of the PBP2‐MreB interaction (using ^malF16^PBP2) resulted in a growth and shape defect, we wondered if the introduction of a mutation enhancing the activity/integrity of the elongasome into the ^*malF16*^ *pbp2‐rodA* construct (either in PBP2 or in RodA) could suppress these deficiencies. Interestingly, introduction of any one of the tested PBP2 or RodA mutations could restore cell growth and rod shape (Figure 2D,E). This suggests that the PBP2‐MreB interaction is critical for cell growth and shape maintenance in wild‐type cells, but it becomes dispensable when the activity or integrity of the elongasome is enhanced by PBP2 or RodA mutations.

**Figure 3:**
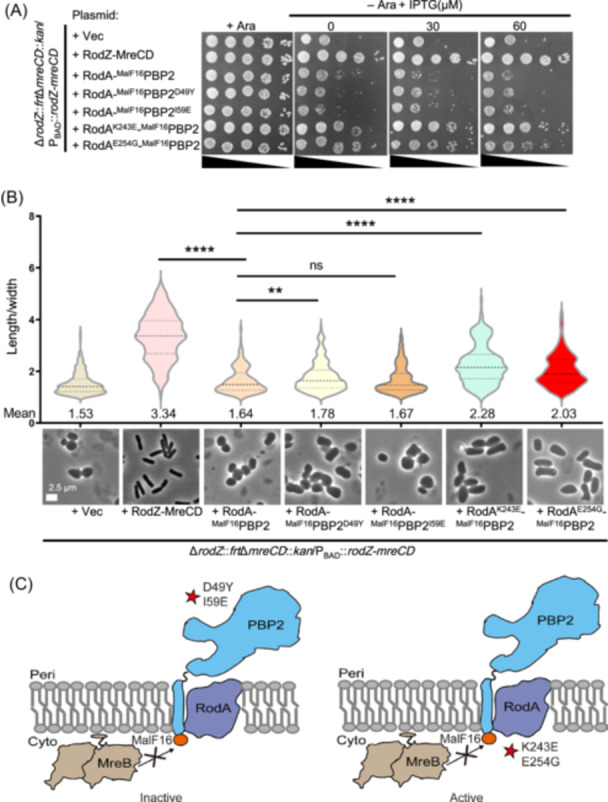
RodA mutations suppress the growth and shape defects caused by ^MalF16^PBP2 in RodZ‐ and MreCD‐depleted cells. (A, B) RodA variants partially suppressed the growth (A) and shape (B) defects caused by ^MalF16^PBP2. Plasmids pDSW210, pZR187, pSD314‐MalF16, or its derivatives were transformed into strain RZ96A on LB plates with ampicillin, chloramphenicol, and 0.2% arabinose at 37°C. The spot test was performed as in Figure 1A. To examine the cell morphology (B), overnight cultures of the strains from (A) were diluted 1:100 in LB medium with antibiotics and grown at 37°C for 2 h. Cells were fixed and imaged. Cell aspect ratios (length/width) were analyzed with Image J. Data are shown as the mean ± SD. Number of cells analyzed is more than 200. Statistical significance was assessed by unpaired *t*‐test with Welch's correction. **p < 0.01; ****p < 0.0001. ns, not significant. Scale bar, 2.5 µm. (C) Schematic depicting the effects of disruption of the PBP2‐MreB interaction on the function of an elongasome lacking RodZ and MreCD. Left: PBP2 mutations fail to suppress the growth and shape defects caused by ^MalF16^PBP2. Right: RodA mutations can partially correct the growth and shape defects caused by ^MalF16^PBP2 in the absence of MreCD and RodZ.

To further test if the PBP2‐MreB interaction becomes important for the function of the minimal elongasome, which contains only MreB and the mutant RodA‐PBP2 complex, plasmids with various ^*malF16*^ *pbp2‐rodA* alleles were introduced into the RodZ‐MreCD depletion strain RZ96A. The resulting strains were tested on LB plates without arabinose and in the presence of IPTG at 37°C. As shown in Figure 3A,B, ectopic expression of RodA‐^MalF16^PBP2^D49Y^ or RodA‐^MalF16^PBP2^I59E^ failed to rescue the growth and the shape defects of the RodZ‐MreCD‐depleted cells. Intriguingly, RodA^K243E^‐^MalF16^PBP2 or RodA^E254G^‐^MalF16^PBP2 partially suppressed the growth defects caused by the absence of MreCD and RodZ, as spherical cells were converted into short rods (Figure 3A,B). These results indicate that the PBP2‐MreB interaction is critical for the minimal elongasome to synthesize lateral PG when the mutations are in PBP2 (D49Y and I59E), but is not necessary in the presence of RodA mutations (K243E and E254G). We speculated that the RodA‐PBP2 complex interacts with MreB largely via the PBP2‐MreB interaction when the mutations are in PBP2. As a consequence, disruption of the PBP2‐MreB interaction abolishes the ability of PBP2 mutations to suppress the defects caused by depletion of MreCD and RodZ. However, in the presence of RodA mutations, the complex interacts with MreB largely through an interaction between MreB and RodA, such that disruption of the PBP2‐MreB interaction does not eliminate the ability to rescue the defects of MreCD‐ and RodZ‐depleted cells. Collectively, these results suggest that the PBP2‐MreB interaction plays an important role in connecting MreB to the RodA‐PBP2 complex (driven by PBP2 mutations) for lateral PG synthesis in the absence of MreCD and RodZ.

### The C‐terminal tail of RodA interacts with MreB and is important for elongasome function

**Figure 4:**
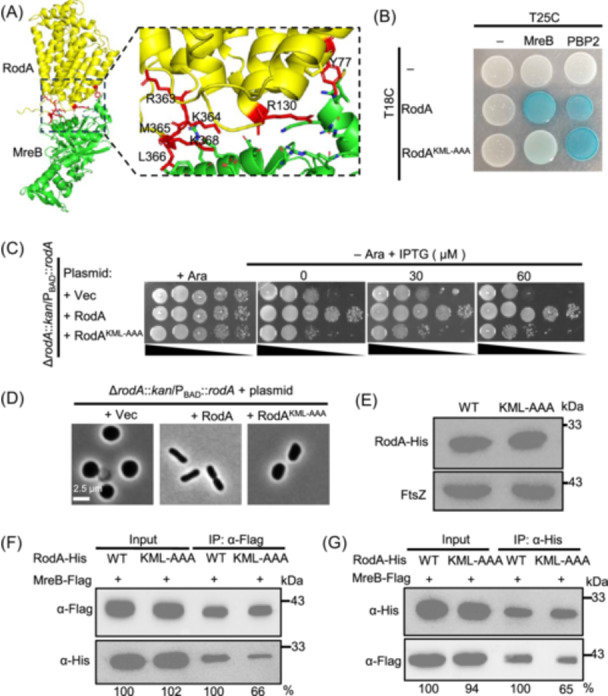
The C‐terminal tail of RodA mediates its interaction with MreB. (A) A structural model of the *E. coli* RodA‐MreB complex. The model was extracted from a structural model of the complete elongasome complex generated with AF3. Residues on RodA predicted to be involved in its interaction with MreB are shown in stick form and colored red. (B) Examination of the RodA–MreB interaction by the BTH assay. Pairs of plasmids harboring *mreB′‐t25c‐mreB′* and *t18c‐rodA* or its variant were co‐transformed into BTH101. BTH assays were performed as in Figure 2B. (C) RodA^KML‐AAA^ failed to complement a RodA depletion strain. Plasmid pDSW210, pZR161, or its derivatives carrying different alleles of *rodA* were transformed into strain SD432 on LB plates with ampicillin, chloramphenicol, and 0.2% arabinose at 37°C. The spot test was performed as in Figure 1A. (D) RodA^KML‐AAA^ failed to restore cell shape in RodA‐depleted cells. Overnight cultures of the strains from (B) were diluted 1:100 in LB medium with antibiotics and grown at 37°C for 2 h. These arabinose‐free cultures were then diluted 1:20 in fresh LB medium with antibiotics and 60 μM IPTG, and grown at 37°C for 3–4 h. Cells were fixed on 2% agarose pads for photography. Scale bar, 2.5 µm. (E) Immunoblot to detect the protein level of the RodA variant. Cells were collected, resuspended in SDS‐PAGE sample buffer, and kept at room temperature for 30 min before they were loaded on the SDS‐PAGE gel for analysis. (F–G) Co‐IP analysis of the interaction between MreB‐Flag and RodA‐His. Overnight cultures of TB28 carrying pZR318 (P_206_::*mreB‐flag*‐*rodA‐*6× *his*) and pZR318‐KML (P_206_::*mreB‐flag*‐*rodA* ^*KML‐AAA*^ *‐*6× *his*) were diluted 1:100 in 50 ml of fresh LB medium with antibiotics and 60 μM IPTG, and grown at 37°C for 3 h. Samples were prepared as in Figure 2D. Immunoprecipitation was performed using the anti‐Flag antibody and the anti‐His antibody. The intensity of each band was quantified by ImageJ. Data are representative of two independent biological replicates.

In the AF3 model of the elongasome complex, the cytoplasmic loops and the C‐terminal tail of RodA interact with MreB (Figure 4A). To test the interaction, we first conducted a co‐IP experiment using cells expressing Flag‐tagged MreB (MreB‐Flag) and 6× His‐tagged RodA (RodA‐His). As shown in Figure S7A,B, a band matching the size of RodA‐His was detected in immunocomplexes isolated with the anti‐Flag antibody in cells expressing both MreB‐Flag and RodA‐His, and *vice versa*. Consistent with this, the BTH assay also showed that RodA interacted strongly with MreB (Figure 4B). To determine the regions of RodA mediating its interaction with MreB, we mutated the potential residues in RodA predicted by the AF3 model (Figure S4) and tested if they affect RodA function using a complementation test. As shown in Figure S8A, while single mutations Y77A and K130A did not affect the ability of RodA to complement the RodA depletion strain, single or double mutations in the C‐terminal tail of RodA (K364A, M365A: KM‐AA; L366A; and K368A, S369A: KS‐AA) could not fully rescue the growth defect of RodA‐depleted cells. These results suggest that the C‐terminal tail of RodA, but not the cytoplasmic loops, mediates interaction with MreB. Therefore, we constructed a triple‐mutation variant RodA^K364A, M365A, L366A^ (RodA^KML‐AAA^), and found that it was unable to complement the RodA depletion strain (Figure 4C,D). Immunoblotting experiments showed that the expression level of 6× His‐tagged RodA^KML‐AAA^ was comparable to that of wild‐type RodA‐His (Figure 4E), arguing that the mutations did not affect its stability. More importantly, introduction of the KML‐AAA triple mutations into RodA‐His strongly reduced its association with MreB in wild‐type cells, as detected by co‐IP using anti‐His or anti‐Flag antibodies (Figure 4F,G). The same phenomenon was observed in a RodZ‐MreCD depletion strain (Figure S8D). Also, RodA^KML‐AAA^ displayed a decreased interaction with MreB in the BTH assay when compared to wild‐type RodA (Figure 4B), suggesting that these mutations disrupt the RodA‐MreB interaction.

To test the putative interaction site on MreB, we introduced mutations into MreB and tested if they affected MreB function. However, none of the single‐substitution mutants displayed a reduced ability to complement the MreB(CD) depletion strain when expressed from a plasmid in tandem with MreCD (Figure S9). It is possible that a single substitution in MreB is not disruptive enough to reduce its interaction with RodA, or the predicted interaction site on MreB is not accurate. Nonetheless, the above results strongly indicated that RodA interacts with MreB via its C‐terminal tail, consistent with prior fluorescence resonance energy transfer (FRET) results showing a direct MreB‐RodA association^27^.

### The RodA‐MreB interaction is important for lateral PG synthesis by a minimal elongasome

Since our mutations in RodA or PBP2 can suppress the deficiency caused by ^MalF16^PBP2, which is deficient in interaction with MreB, we tested if such mutations could also suppress the defects caused by RodA^KML‐AAA^. The addition of the K243E or E254G mutation to RodA^KML‐AAA^ could complement the RodA depletion strain (Figure S8B). PBP2 mutants, PBP2^D49Y^ or PBP2^I59E^, expressed in tandem with RodA^KML‐AAA^, also rescued the growth and corrected the shape defects caused by RodA depletion (Figure S8C‐D). Thus, the RodA‐MreB interaction is critical for cell survival and cell shape in wild‐type cells, but the defects caused by RodA^KML‐AAA^ could be relieved by our RodA or PBP2 mutants that enhance the integrity of the elongasome.

**Figure 5:**
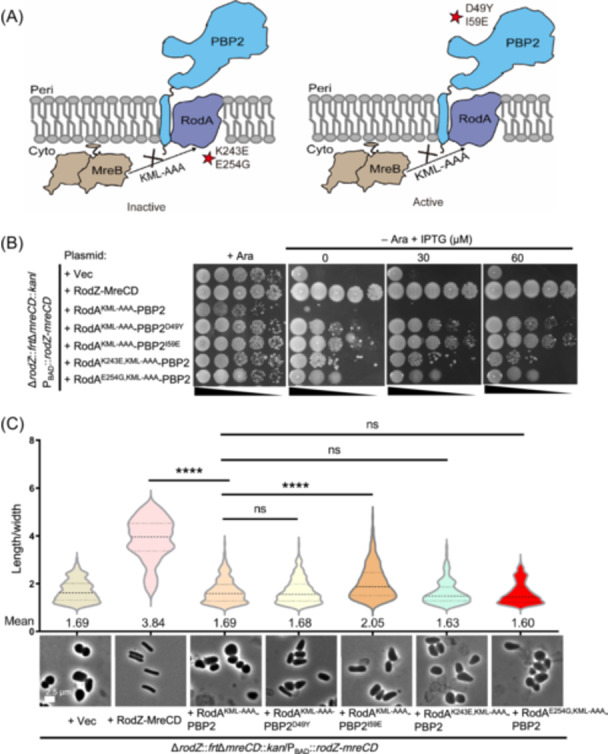
PBP2 mutations suppress the growth and shape defects caused by RodA^KML‐AAA^ in RodZ and MreCD depletion strain. (A) Schematic depicting the effects of the RodA^KML‐AAA^ mutation on the activity of the minimal elongasome. Left: RodA mutations do not rescue the growth and shape defects caused by RodA^KML‐AAA^ in the absence of RodZ and MreCD. Right: PBP2^D49Y^ and PBP2^I59E^ partially suppress the defects caused by RodA^KML‐AAA^. (B, C) PBP2 mutations partially suppressed the growth (B) and shape (C) defects caused by RodA^KML‐AAA^ in the absence of RodZ and MreCD. Plasmid pDSW210, pZR187, pSD314‐KML‐AAA, or its derivatives were transformed into strain RZ96A on LB plates with 0.2% arabinose at 37°C. The spot test was performed as in Figure 1A. To examine cell morphology (C), overnight cultures of the strains from (B) were diluted 1:100 in LB medium with antibiotics and grown at 37°C for 2 h; then, the sample preparation was carried out as described in Figure 1B. Quantification and statistical analysis were performed as in Figure 2E. Scale bar, 2.5 µm.

To test if the RodA‐MreB interaction was vital for the function of the minimal elongasome containing only MreB and the RodA‐PBP2 complex, we ectopically expressed RodA^KML‐AAA^‐PBP2 variants harboring our RodA or PBP2 mutations in the RodZ‐MreCD depletion strain to see if they could rescue growth (Figure 5A). We expected that when the RodA‐MreB interaction was disrupted, the RodA mutations (K243E and E254G) would no longer rescue the growth of the RodZ‐MreCD depletion strain. However, as MreB can still associate with the complex via the PBP2‐MreB interaction, disruption of the RodA‐MreB interaction may not affect the ability of PBP2 mutants (D49Y and I59E) to rescue the RodZ‐MreCD depletion strain. Indeed, ectopic expression of RodA^K243E, KML‐AAA^‐PBP2, or RodA^E254G, KML‐AAA^‐PBP2 was incapable of suppressing the shape and viability defects of the RodZ‐MreCD depletion strain (Figure 5B,C). However, ectopic expression of RodA^KML‐AAA^‐PBP2^D49Y^ or RodA^KML‐AAA^‐PBP2^I59E^ could still ameliorate the growth and shape defects of RodZ‐MreCD‐depleted cells (Figure 5B,C). These results suggest that disruption of the RodA‐MreB interaction abolishes the ability of RodA variants to enhance elongasome activity; however, PBP2 variants could still contact MreB to form a functional minimal elongasome in the absence of the RodA‐MreB interaction (Figure 5A).

### Disruption of both the PBP2‐MreB interaction and the RodA‐MreB interaction inactivates the minimal elongasome

**Figure 6:**
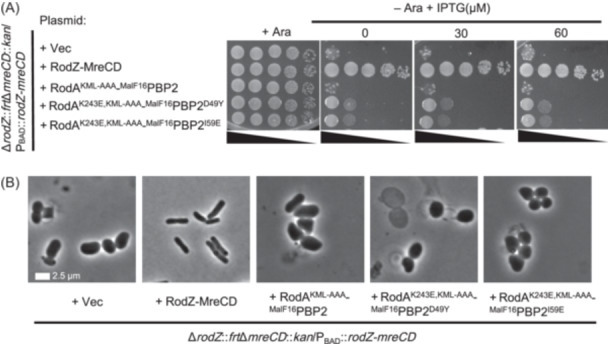
Disruption of the interaction between MreB and RodA‐PBP2 eliminated the ability of a mutant RodA‐PBP2 complex to suppress the growth (A) and shape (B) defects caused by the absence of RodZ and MreCD. Plasmid pDSW210, pZR187, pSD314‐MalF16‐KML(^MalF16^PBP2‐RodA^KML‐AAA^), and its derivatives with different alleles of *rodA‐pbp2* were transformed into strain RZ96A on LB plates with 0.2% arabinose at 37°C. The spot test was performed as in Figure 1A. Samples were prepared as described in Figure 1B. Scale bar, 2.5 µm.

The above results suggest that MreB association with the RodA‐PBP2 complex can occur via either the MreB‐PBP2 interaction or the MreB‐RodA interaction. If so, disruption of both the PBP2‐MreB and RodA‐MreB interactions may be necessary to disconnect MreB from a RodA‐PBP2 complex containing mutations in both proteins. To test this, plasmids with the indicated *rodA‐pbp2* alleles were introduced into the RodZ‐MreCD depletion strain RZ96A, and the resulting strains were tested on LB plates with IPTG at 37°C. As expected, the double mutant (RodA^KML‐AAA^‐^MalF16^ PBP2) containing our mutations in PBP2 or RodA failed to support the growth of RZ96A on LB plates without arabinose (Figure 6A,B). These results indicate a critical role for the PBP2‐MreB and RodA‐MreB interactions to allow an activated elongasome to promote lateral PG synthesis when MreCD and RodZ are absent.

## DISCUSSION

A complete elongasome is necessary for efficient lateral PG synthesis and rod shape maintenance in wild‐type *E. coli* grown on LB. However, a previous study showed that MreB and an activated RodA‐PBP2 complex can partially maintain lateral PG synthesis in the absence of MreCD and RodZ^35^. This finding indicates that MreB must directly engage with an activated RodA‐PBP2 complex to direct lateral PG synthesis, but the exact mechanism is not clear. In this study, we first isolated additional mutations in RodA and PBP2 that partially suppress the growth and shape defects caused by MreCD and RodZ depletion. Using these mutants along with the AF3 structural model of the entire elongasome, we found that both the cytoplasmic N‐terminal region of PBP2 and the C‐terminal tail of RodA interact with MreB to suppress the loss of MreCD and RodZ. Moreover, these interactions are important for elongasome activity in wild‐type cells. These findings reveal the coupling mechanism between the cytoskeleton (MreB) and PG synthetic enzymes (RodA‐PBP2), and the importance of this linkage in driving cell wall synthesis in bacteria.

Variants of elongasome components that enhance elongasome activity or integrity are powerful tools to study the working mechanism of the complex^35^, ^40^. Previous studies revealed that the periplasmic interaction interface of RodA and PBP2 plays a critical role in regulating RodA‐PBP2 activity, likely mediating activating signals coming from MreCD and RodZ^9^, ^35^, ^40^. In this study, we identified additional mutations in this interface that either enhance or disrupt RodA‐PBP2 activity. The RodA and PBP2 mutations that allow the bypass of MreCD and RodZ likely induce a conformational change in the complex, mimicking the action of RodZ and MreCD, resulting in the activation of the PGTase activity of RodA (Figure 1). Similar mutations have also been reported in the periplasmic interaction interface of the FtsW‐FtsI complex, a paralogous SEDS‐bPBP complex responsible for sPG synthesis during cell division^10^, ^34^, ^42^, ^43^. These mutations enable the cells to divide in the absence of the constriction trigger protein FtsN, or in the presence of inactive mutations in the regulatory subcomplex FtsQLB^34^, ^44^. These findings underscore the critical role of the interaction between the ECL4 of SEDS proteins and cognate bPBPs′ hinge and pedestal domains for regulating the activity of these complexes^9^, ^34^, ^37^. More importantly, they suggest that analogous mechanisms control lateral PG synthesis in cell elongation and sPG synthesis in cell division.

The isolation of mutations enhancing elongasome activity in RodA and PBP2 enabled us to explore how the RodA‐PBP2 complex interacts with MreB to carry out lateral PG synthesis in the absence of MreCD and RodZ. Co‐IP analysis and BTH assays showed that PBP2 interacts with MreB via its N‐terminal cytoplasmic domain, and the absence of this domain (^MalF16^PBP2) caused shape and growth defects (Figure 2B,C). These results suggest that the PBP2‐MreB interaction is critical for lateral PG synthesis in wild‐type cells. More importantly, disruption of the PBP2‐MreB interaction (by ^MalF16^PBP2) abolished the ability of PBP2 variants (PBP2^D49Y^ and PBP2^I59E^) to suppress the growth defects of RodZ‐MreCD‐depleted cells (Figure 3A). A previous report suggested that the cytoplasmic domain of PBP2 affects MreCD‐mediated regulation of the interaction between RodA and PBP2^27^. However, we found that ^MalF16^PBP2 interacted with RodA as well as wild‐type PBP2 in the BTH assay. Based on our results, we believe that the main function of the cytoplasmic domain of PBP2 is to interact with MreB. This PBP2‐MreB interaction is important for elongasome activity in wild‐type cells, but it becomes dispensable when mutations are present in PBP2 or RodA to increase the activity or integrity of the elongasome. This might be because the mutant RodA‐PBP2 complex interacts with other elongasome components better than the wild‐type RodA‐PBP2, such that this deficiency is compensated for. Nonetheless, in the absence of MreCD and RodZ, the PBP2‐MreB interaction becomes critical for PBP2 variants to engage with MreB.

In addition to the PBP2‐MreB interaction, our results suggest that MreB can also associate with the RodA‐PBP2 complex via an interaction with the C‐terminal tail of RodA. The RodA^KML‐AAA^ variant showed reduced interaction with MreB, as revealed by the co‐IP test and the BTH assay (Figure 4B,E,F). Importantly, it failed to complement a RodA depletion strain (Figure 4C,D). However, adding RodA or PBP2 mutations that enhance the elongasome function to RodA^KML‐AAA^ allowed rescue, suggesting that the RodA‐MreB interaction becomes dispensable, similar to the situation for the PBP2‐MreB interaction. Nonetheless, disruption of the RodA‐MreB interaction by the RodA^KML‐AAA^ mutation eliminated the ability of RodA mutants, but not PBP2 mutants, to support cell growth and restore rod shape in cells lacking MreCD and RodZ (Figure 5B,C). These observations suggest that MreB can engage with the RodA‐PBP2 complex either via an interaction between MreB and RodA or an interaction between MreB and PBP2. In line with this, co‐IP tests showed that disruption of the PBP2‐MreB or RodA‐MreB interaction could not completely eliminate the association of MreB with the RodA‐PBP2 complex. Furthermore, these observations also suggest that mutations in PBP2 or RodA not only affect the activity of the RodA‐PBP2 complex but also influence the ability of the complex to interact with other elongasome components.

A similar scenario has been reported with the divisome complex. FtsA, an MreB‐like protein, interacts with FtsW (a paralog of RodA) to activate and organize sPG synthesis. Mutations (Y163K and R172D) in intracellular loops 2 and 4 of FtsW reduced its interaction with FtsA, resulting in cell division block^45^. However, the defect caused by these mutations could be suppressed by an activating FtsW mutation (E289G). These observations suggest that in both the elongasome and the divisome of *E. coli*, the cytoskeletal protein MreB/FtsA needs to engage with the SEDSbPBP complex, RodA‐PBP2, or FtsW‐FtsI to organize PG synthesis.

Although our results suggest that MreB can direct the mutant RodA‐PBP2 complex to the right places for lateral PG synthesis via either the PBP2‐MreB or RodA‐MreB interaction, MreCD and RodZ are critical for lateral PG synthesis in wild‐type cells in many bacteria. How they regulate elongasome activity remains to be elucidated. An *rodZ* deletion results in aberrant assembly of MreB as well as other elongasome components^15^, ^16^. However, mutations in MreB, RodA, PBP2, MreC, and MreD (presumably increasing the activity or integrity of elongasome) can largely restore growth and rod shape to *rodZ* mutant cells^35^, ^41^, ^46^, suggesting restoration of proper elongasome assembly. Under A22 treatment, when the localization of MreB becomes evenly distributed in the cytoplasm, the typical spotty membrane localization of MreB was maintained in the presence of such suppressor mutations. Rohs et al. showed that the activating mutation PBP2^L61R^ increased the number of directionally moving MreB filaments per cell^35^. Also, the MreB filaments observed in PBP2^L61R^ cells were on average significantly shorter than those found in PBP2 wild‐type cells. Based on these observations, they suggested that MreB polymerization is modulated by the activation status of the RodA‐PBP2 synthase^35^. These findings indicate that the localization and assembly of MreB are not only regulated by RodZ but also by active lateral PG synthesis by RodA‐PBP2. Intriguingly, some rod‐shaped bacteria do not encode RodZ, such as *Helicobacter pylori* ^39^, implying that these bacteria may regulate lateral PG synthesis by distinct mechanisms. One possibility is that the RodA‐PBP2 complex in these bacteria is naturally active so that it can strongly associate with MreB to synthesize lateral PG in the absence of RodZ. However, future investigations are necessary to uncover the mechanisms governing the activity of these modified elongasomes.

In summary, we show that MreB directly interacts with the RodA‐PBP2 complex to organize lateral PG synthesis in *E. coli*. Our work shows that these interactions are not only important for presumed activation mutations in PBP2 and RodA to rescue cells lacking RodZ and MreCD but also in wild‐type cells. This study expands our understanding of the working mechanism of the elongasome and will facilitate the reconstruction of lateral PG synthesis *in vitro* and screening for its inhibitors.

## MATERIALS AND METHODS

### Media, bacterial strains, plasmids, and growth conditions

The strains generated and used in this study are derivatives of MG1655 or W3110 and cultured in LB medium (1% tryptone, 0.5% yeast extract, 0.5% NaCl, and 0.05 g/l thymine). Antibiotics were used at the following concentrations when necessary: 100 μg/ml ampicillin (Amp); 25 μg/ml spectinomycin (Spc); 25 μg/ml kanamycin (Kan); 12.5 μg/ml tetracycline (Tet); and 15 μg/ml chloramphenicol (Chl). The strains and plasmids used in this study are listed in Tables S1 and S2, respectively. Construction of strains and plasmids is described in the Supporting Information section, with the primers listed in Table S3.

### Mutagenesis

Mutations in indicated genes were introduced by site‐directed mutagenesis using the Quickchange II kit (Agilent) or by overlap PCR. The primer pairs used for mutagenesis are provided in Table S3. All mutations were confirmed by sequencing.

### Allelic replacement in *E. coli*

Different alleles of *pbp2* or *rodA* were introduced into the chromosome at its native locus by allelic replacement as described by Hamilton et al.^47^ The principle of allelic replacement was achieved through homologous recombination between a chromosomal target locus and the mutant alleles on a plasmid, which is temperature senstive for replication. The mutations were first introduced into a plasmid (pSC101^ts^, *rodA‐pbp2*) by site‐directed mutagenesis or overlap PCR. These plasmids were transformed into the wild‐type strain TB28 and selected on LB plates containing spectinomycin at 44°C. Because the plasmids replicate at 30°C, but not at 44°C, they integrated into the gene of interest, resulting in integrants carrying the mutations. Subsequently, these co‐integrants were grown at 30°C to trigger a second recombination event, causing the excision of the plasmids. The second recombination event led to a gene replacement or retention of the original copy of the gene. 10–20 colonies were randomly picked and the *pbp2* or *rodA* gene from these colonies was sequenced to detect whether the mutation was successfully introduced into the chromosome.

### Western blot

To measure the protein level of elongasome protein and its mutants, overnight cultures of TB28 (wild‐type strain) expressing the indicated proteins were diluted 1:100 in LB medium with ampicillin and 50 μM IPTG. After growth at 37°C for 2 h, samples were then taken and adjusted according to OD_600_ of the cultures to ensure that an equal amount of cells was used for each strain. Cells were collected, resuspended in SDS‐PAGE sample buffer, and boiled for 10 min (PBP2) or kept at room temperature for 30 min (RodA), before they were taken for analysis by SDS‐PAGE. The anti‐GFP antibody (HT801; TRANSGEN) and the anti‐Flag (66008‐4‐lg; Proteintech Group) antibody were used at a dilution of 1/10,000. The anti‐His (66005‐1‐lg; Proteintech Group) antibody was used at a dilution of 1/5000.

### Immunoprecipitation

Overnight cultures of strains carrying the indicated expression plasmids were diluted 1:100 in 50 ml of fresh LB medium and cultivated at 37°C until OD_600_ reached 0.4–0.6. Cells were harvested by centrifugation at 12,000 rpm for 10 min, resuspended in 1.5 ml PBST (1× PBS with 0.5% Tween‐20) buffer containing a 1% antiprotease cocktail (MCE), and lysed by sonication. The lysates were centrifuged at 12,000 rpm for 5 min to remove cell debris. 400 μl of the supernatant was transferred to antibody‐conjugated magnetic beads pre‐equilibrated in PBST and incubated for 8 h at 4°C. The bead–antibody–supernatant complexes were then separated using a magnetic rack and washed four times with 400 μl of PBST. Immunocomplexes were ultimately eluted by resuspending the bead pellets with SDS‐PAGE sample buffer by heating at 95°C for 10 min. The eluates were then resolved by SDS‐PAGE and subjected to Western blotting analysis.

### Spot assay for the A22 resistance test

To test the sensitivity of *E. coli* RodA variants or PBP2 variants to A22, plasmid pZR161 (pDSW210, P_206_::*rodA*) or its derivatives, and pSD314 (pDSW210, P_206_::*pbp2‐rodA*) or its derivative vectors, were transformed into strain SD432 (W3110, Δ*rodA*::*kan*/pSD308 (P_BAD_::*rodA*)) and strain RZ50 (TB28, Δ*(pbp2‐rodA)*::*kan*/pSD312(P_BAD_::*pbp2‐rodA*)), respectively. The resultant strains expressing indicated alleles of *rodA* or *pbp2* were cultured in LB medium. The next day, a single transformant of the resulting strains was resuspended in 1 ml of LB medium and serially diluted in 10‐fold. 2.5 μl of each dilution was spotted on LB plates with appropriate antibiotics; IPTG and A22 were added to the culture to a final concentration as indicated.

### BTH assay

To detect the interaction between elongasome proteins or its mutants, the corresponding plasmid pairs were co‐transformed into BTH101. Transformants were selected on plates with antibiotics and glucose overnight at 37°C. The next day, single colonies were resuspended in 1 ml of LB medium, and 2.5 μl of each aliquot was spotted on LB plates containing 100 μg/ml ampicillin, 25 µg/ml kanamycin, 40 μg/ml X‐gal, and the indicated concentration of IPTG. Plates were incubated at 30°C for 16 h and photographed.

### Microscopy

Phase contrast and epifluorescence images were acquired on an Olympus BX53 upright microscope equipped with a Retiga R1 CCD camera (QImaging), a CoolLED pE‐4000 illumination system, and a U Plan XApochromat phase‐contrast objective lens (100×, 1.45 numerical aperture [NA], oil immersion). Images were captured and processed using VisionView software.

To investigate the impact of an active RodA‐PBP2 mutation on restoration of shape in the MreCD and RodZ depletion strain, overnight cultures of RZ96A (TB28, Δ*mreCD*::*kan*, Δ*rodZ*::*frt*/pZR186(P_BAD_::*rodZ‐mreCD*)) that harbored a plasmid expressing alleles of *rodA‐pbp2* were diluted 1:100 in fresh LB medium with appropriate antibiotics and 0.2% arabinose, and grown at 37°C for 2 h. Cells were then collected by centrifugation and washed twice with fresh LB to remove arabinose and resuspended in the same volume of LB medium. These cultures were then diluted 1:100 in fresh LB medium with appropriate antibiotics, and grown at 37°C for 2–3 h. The cultures were diluted 1:10 in 5 ml of fresh LB medium again and IPTG was added to a final concentration of 30 μM and grown at 37°C about 3–4 h; cells were immobilized on a 2% agarose pad and photographed.

### Quantification and statistical analysis

Morphometric analyses were performed with ImageJ. Briefly, phase‐contrast images were imported into ImageJ and converted into 8‐bit versions with the scale bar embedded. Each cell was traced with the polygon selection tool and cells that were incompletely captured within the field of view were discarded. The aspect ratio was calculated by dividing the length measurements by the width measurements. A minimum of 200 cells per strain was evaluated. Aspect ratio data were assembled in GraphPad Prism (v.10.0.1), and statistical analysis of the aspect ratio was carried out in GraphPad Prism using a parametric unpaired *t* test assuming a Gaussian distribution but not equal SD (Welch's correction). Intensities of each band from Western blot were also quantified by ImageJ.

## AUTHOR CONTRIBUTIONS

**Rui Zhan**: Data curation; formal analysis; investigation; methodology; validation; visualization; writing—original draft. **Han Gong**: Data curation; investigation; methodology. **Ying Li**: Data curation; investigation; methodology. **Xiangdong Chen**: Resources; supervision. **Joe Lutkenhaus**: Resources; supervision; writing—original draft; writing—review and editing. **Shishen Du**: Conceptualization; data curation; formal analysis; funding acquisition; project administration; resources; supervision; writing—original draft; writing—review and editing.

## ETHICS STATEMENT

This study did not involve human subjects and animals.

## CONFLICT OF INTERESTS

The authors declare no conflict of interests.

## Supporting information

Supplement information.

## Data Availability

All data presented in this study will be publicly available as of the date of publication.
