# Supplementary material for: Direct interaction between MreB and the RodA‐PBP2 complex organizes lateral cell wall synthesis in Escherichia coli
Source: mLife. 2026 May 18;5(3):325–38. doi: 10.1002/mlf2.70079 (PMC13327600; doi:10.1002/mlf2.70079)
Supplement: Supplementary file 1 — Supplement information. [file MLF2-5-325-s001.docx]

**Direct interaction between MreB and the RodA-PBP2 complex organizes lateral cell wall synthesis in *Escherichia coli***

**Authors**

Rui Zhan^1, 2^, Han Gong^1, 2^, Ying Li^1, 2^, Xiangdong Chen^3^, Joe Lutkenhaus^4^, and Shishen Du^1, 2^*

**Affiliation**

1 State Key Laboratory of Metabolism and Regulation in Complex Organisms, College of Life Sciences, Wuhan University, Wuhan, Hubei, China

2 Hubei Key Laboratory of Cell Homeostasis, College of Life Sciences, Wuhan University, Wuhan, Hubei, China

3 State Key Laboratory of Virology and Biosafety, College of Life Sciences, Wuhan University, Wuhan, Hubei, China

4 Department of Microbiology, Molecular Genetics and Immunology, University of Kansas Medical Center, Kansas City, Kansas, USA

*** To whom correspondence should be addressed:**

Shishen Du

State Key Laboratory of Metabolism and Regulation in Complex Organisms,

College of Life Sciences, Wuhan University, Wuhan, Hubei, China

e-mail: [ssdu@whu.edu.cn](mailto:ssdu@whu.edu.cn)

**Supplementary Figure and Legends**

**
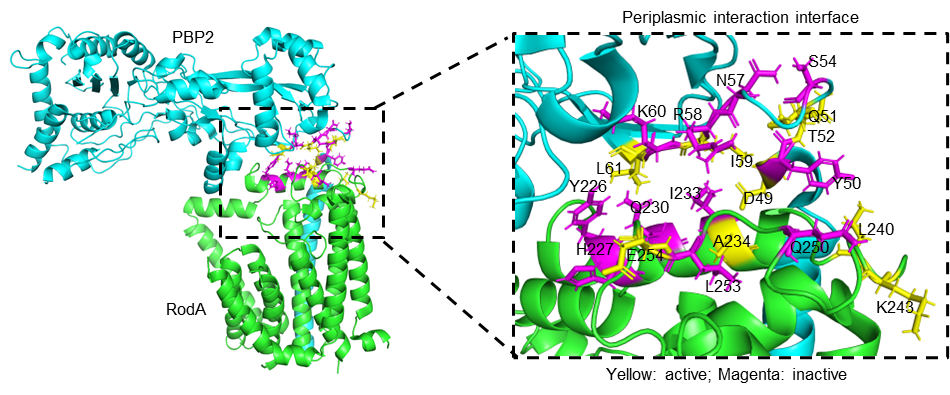
**

**Fig. S1 Locations of residues in *E. coli* RodA-PBP2 complex (PDB ID#: 8TJ3) (1) analyzed in this study.** RodA was colored green and PBP2 was colored cyan. The periplasmic interface between RodA and PBP2 was boxed and enlarged. Residues altered by mutations were indicated. Activating mutations were colored yellow, while inactive mutations were colored magenta.


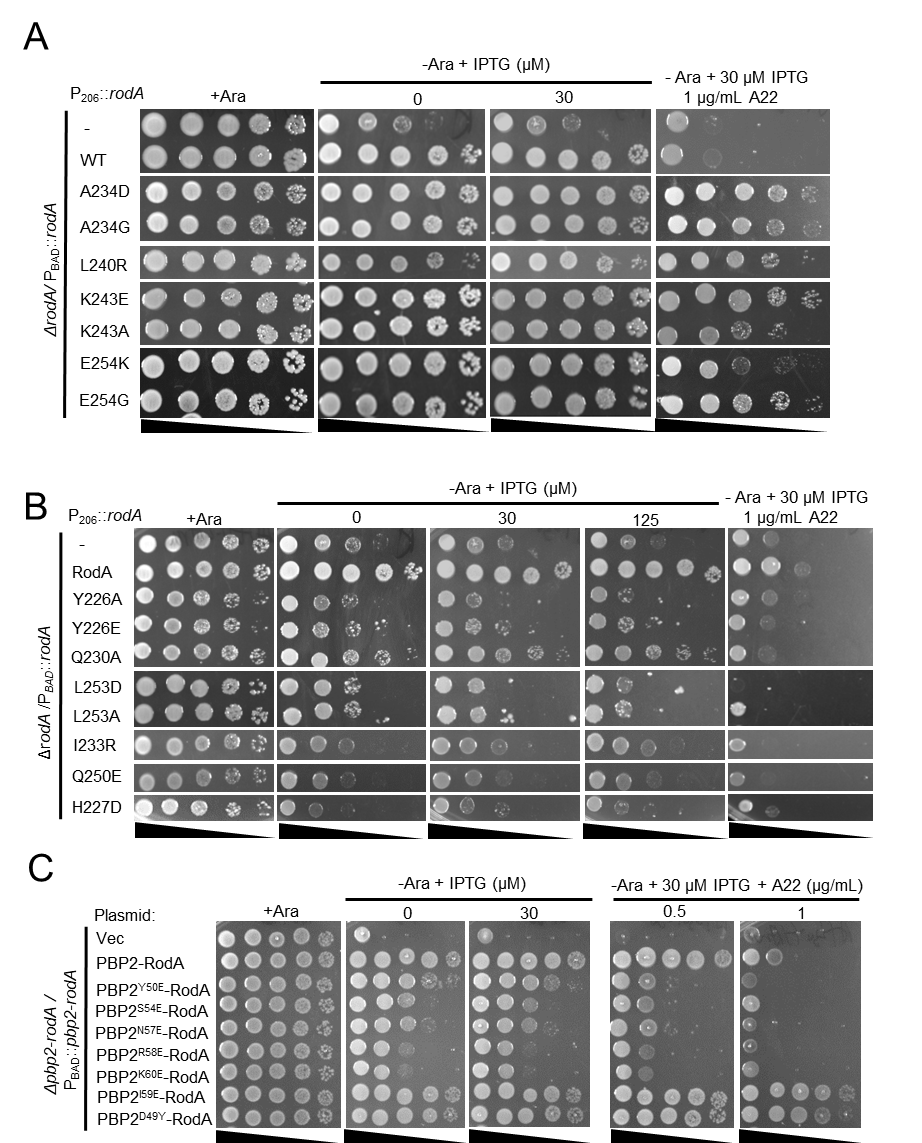


**Fig. S2 Screen for RodA or PBP2 mutations providing resistance to A22.** (A) A spot test of A22 resistant mutations in *rodA*. Plasmid pDSW210, pZR161 (pDSW210, P_206_::*rodA*) or its derivatives with different *rodA* alleles were transformed into strain SD432 (W3110, *rodA*::*kan* / pSD308 (P_BAD_::*rodA*)) and transformants were selected on LB plates with antibiotics and arabinose at 37°C. The next day, a single transformant of the resulting strains were resuspended in 1 mL LB medium and serially diluted in 10-fold. 2.5 μL of each dilution was spotted on LB plates with antibiotics, IPTG and A22 were added to the culture to a final concentration as indicated. Plates were incubated at 37°C overnight and photographed. (B) Complementation test of inactive RodA mutants. Strains containing the plasmid expressing the indicated alleles of *rodA* were grown on LB plates, and the spot test was performed as in (A). (C) Screen for mutations affecting PBP2 function. Plasmid pDSW210, pSD314 (pDSW210, P_206_::*pbp2-rodA*) or its derivatives with different *pbp2* alleles were transformed into strain RZ50 (TB28, *mrdAB*::*kan* / pSD312 (P_BAD_::*pbp2-rodA*)) and transformants selected on LB plates with antibiotics and arabinose at 37°C. IPTG and A22 were added to the culture to a final concentration as indicated, the spot test was performed as in (A).


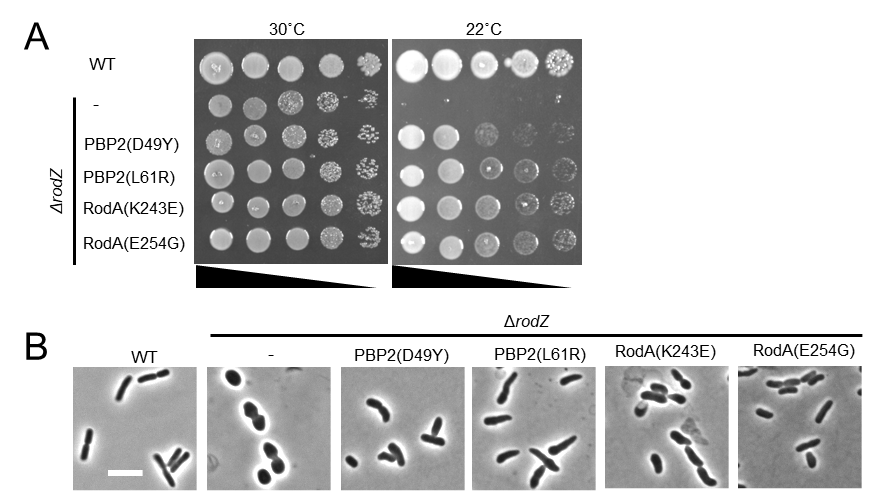


**Fig. S3 A22-resistant RodA or PBP2 mutations suppress the growth and shape defects of Δ*rodZ* cells**. (A) RodA or PBP2 mutations rescued the growth defects caused by *rodZ* deletion. Strains (RZ56 to RZ61) containing the indicated *pbp2 or rodA* alleles at the native genomic loci were serially diluted and spotted on LB plate supplemented with antibiotics. Plates were incubated at 22°C (non-permissive condition) for 40 h or 30°C (permissive condition) for 16 h prior to being photographed. The *pbp2 or rodA* alleles include *pbp2^D49Y^*, *pbp2^L61R^*, *rodA^K243E^ and rodA^E254G^*. (B) RodA or PBP2 mutations rescued the shape defects caused by RodZ deletion. Overnight cultures of the indicated strains (RZ56 to RZ61) were diluted 1:100 in 5 mL LB supplemented with kanamycin and grown at 30°C until an OD_600_ of 0.4 to 0.6. Cells were immobilized on an agarose pad and imaged. (-) denotes the Δ*rodZ* strain with wild type PBP2 and RodA. Scale bar, 5 µm.


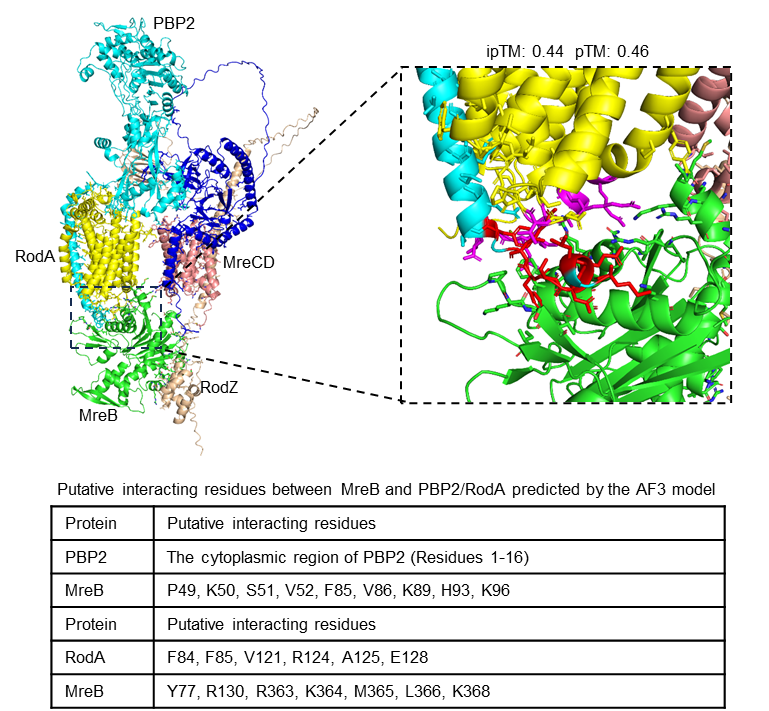


**Fig. S4** **A structural model of the *E. coli* elongasome by AlphaFold 3.** The whole elongasome complex (RodZ-MreB-MreC-MreD-RodA-PBP2) was predicted in a 1:1:1:1:1:1 stoichiometry. Proteins were colored as following: MreB (Green); RodZ (Light brown); RodA (Yellow); PBP2 (Cyan); MreC (Blue); MreD (Salmon). The putative interaction interfaces between MreB and RodA-PBP2 complex were boxed and highlighted. The N-terminal cytoplasmic domain of PBP2 (color red) as well as the cytoplasmic loops and the C-terminal tail of RodA (color magenta) were predicted to contact with MreB. Residues on RodA or PBP2 that were predicted to be involved in interactions were labelled and summarized in the table. Template modeling (pTM) score and the interface predicted template modeling (ipTM) score were marked in the figure. Sequences of elongasome components were obtained from Uniport (https://www.uniprot.org/)：*Escherichia coli* PBP2 (P0AD65)，RodA ([P0ABG7](https://www.uniprot.org/uniprotkb/P0ABG7/entry)), MreB ([P0A9X4](https://www.uniprot.org/uniprotkb/P0A9X4/entry)), [MreC (](https://www.uniprot.org/uniprotkb/Q01466/entry)P16926), MreD (P0ABH4), RodZ (P27434). Structural Prediction Website: <https://alphafoldserver.com/>.

**
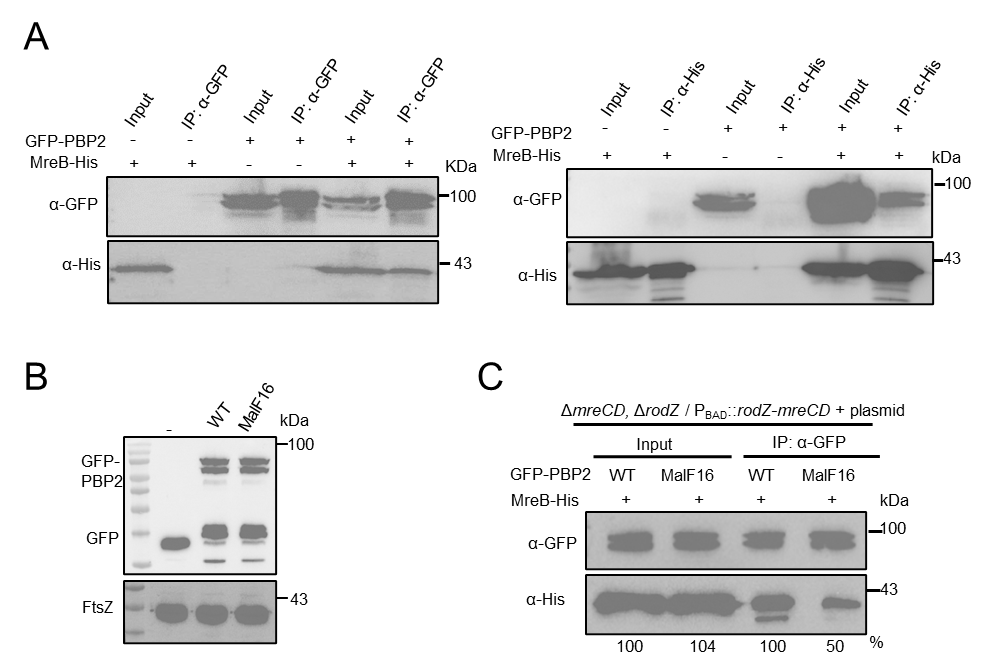
Fig. S****5 Analysis of the interaction between PBP2 and MreB and stability of PBP2 mutant.** (A) PBP2 interacted with MreB as detected by Co-IP. Overnight cultures of TB28 carrying the pZR288, pZR219 or both plasmids were diluted 1:100 in 50 mL fresh LB medium with antibiotics and 60 μM IPTG, and grown at 37°C for 3 hours. The details of SDS-PAGE sample preparation were described in Methods. (B) Western blot to determine the protein level of PBP2 variant. Overnight cultures of TB28 harboring the plasmids pZR180 or its derivative with *gfp-^malF16^pbp2-rodA* were diluted 1:100 in LB medium with ampicillin and 60 μM IPTG. After growth about 2-3 h at 37°C , OD_600_ of each culture was measured and samples were taken for western blot. Cells were collected, resuspended in SDS-PAGE sample buffer and boiled for 10 min at 95°C before they were loaded on the SDS-PAGE gel for analysis. FtsZ was used as a loading control. Anti-GFP and Anti-FtsZ antibody was used at a dilution of 1/10,000. (C) ^MalF16^PBP2 displayed reduced interaction with MreB in RodZ-MreCD depleted cells as detected by Co-IP. The details of SDS-PAGE sample preparation were described in Methods. Intensitiy of each band was quantitated by Image J.

**
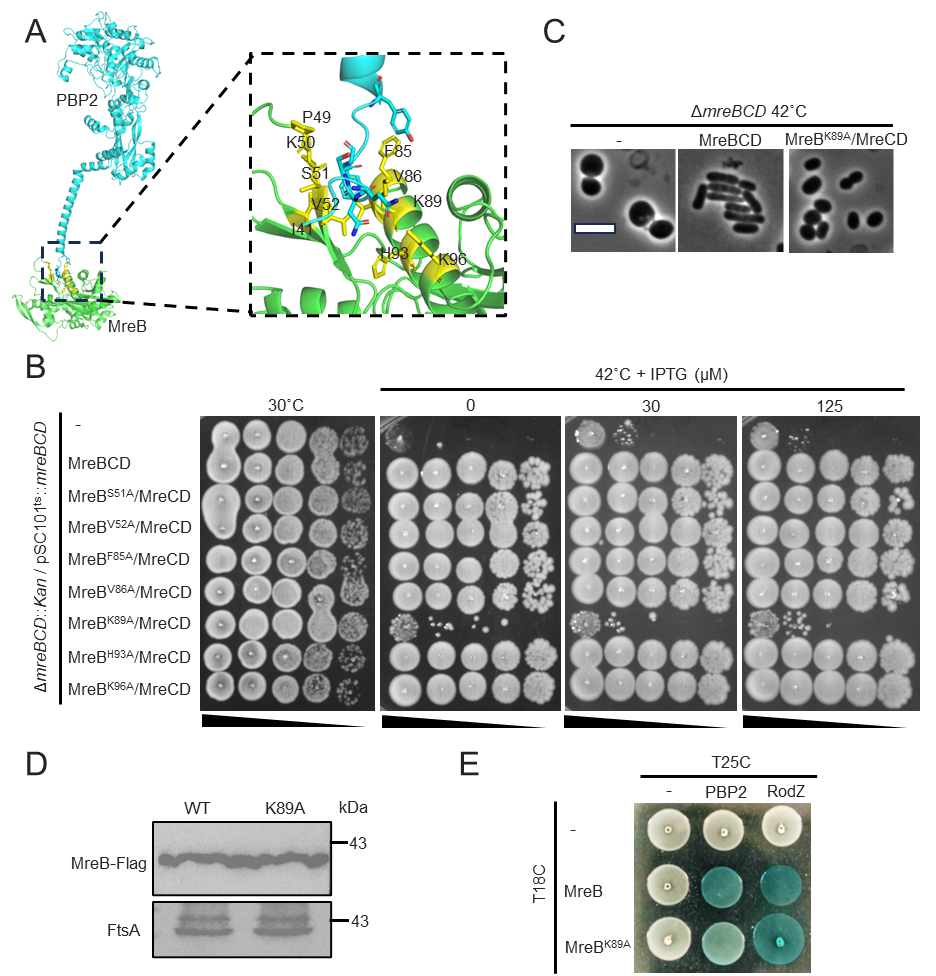
**

**Fig. S6 Residue K89 of MreB is important for its interaction with PBP2.** (A) A structural model of the *E. coli* MreB-PBP2 complex obtained with AlphaFold3. The model was extracted from a structural model of the complete elongasome complex generated by AF3 (Fig. S4). Residues on MreB predicted to be involved in its interaction with PBP2 were shown in stick, colored red. (B) Complementation test of MreB mutants. Plasmid pDSW210, pSD316 or its derivatives with different *mreB* alleles were transformed into strain RZ49 and transformants were selected on LB plates with antibiotics and arabinose at 30°C. The spot test was performed as in Fig. 1A. Plates were incubated at 30°C or 42°C overnight and photographed. (C) Morphology of MreBCD depleted cells complemented with wild type or MreBCD mutants. Overnight cultures of the strains from panel (B) were cultured, grown and immobilized on 2% agarose pads for photography as described in Fig. 1B . Scale bar, 5 µm. (D) Western blotting to determine the protein level of wild type or mutant MreB. The test was performed as in Fig. 4E. (E) Bacterial two hybrid test of the interaction between PBP2 and MreB mutant. Pairs of plasmids harboring *mreB’-t18C-mreB’* and *t25c-pbp2* or it variants were co-transformed into BTH101. The spot test was performed as in Fig. 2B.

**
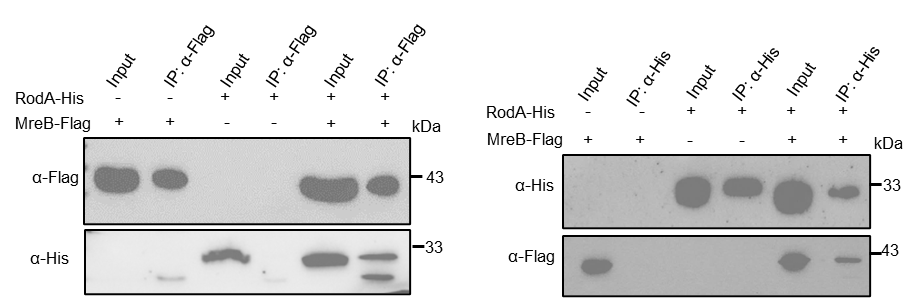
**

**Fig. S7 Co-IP analysis of the interaction between MreB and RodA.** Overnight cultures of TB28 carrying pZR313, pZR314 or pZR318 were diluted 1:100 in 50 mL fresh LB medium with antibiotics and 60 μM IPTG, and grown for 3 h at 37°C . Cells were collected and lysed by sonication and then centrifuged at 12,000 rpm for 10 min at 4°C to remove cell debris. Supernatants were incubated with antibodies coated magnetic beads. Immunocomplexes were collected, resuspended in SDS-PAGE sample buffer and kept at room temperature for 30 min before they were loaded on the SDS-PAGE gel for Western blot analysis. Anti-His and Anti-Flag antibodies were used at a dilution of 1/10,000.


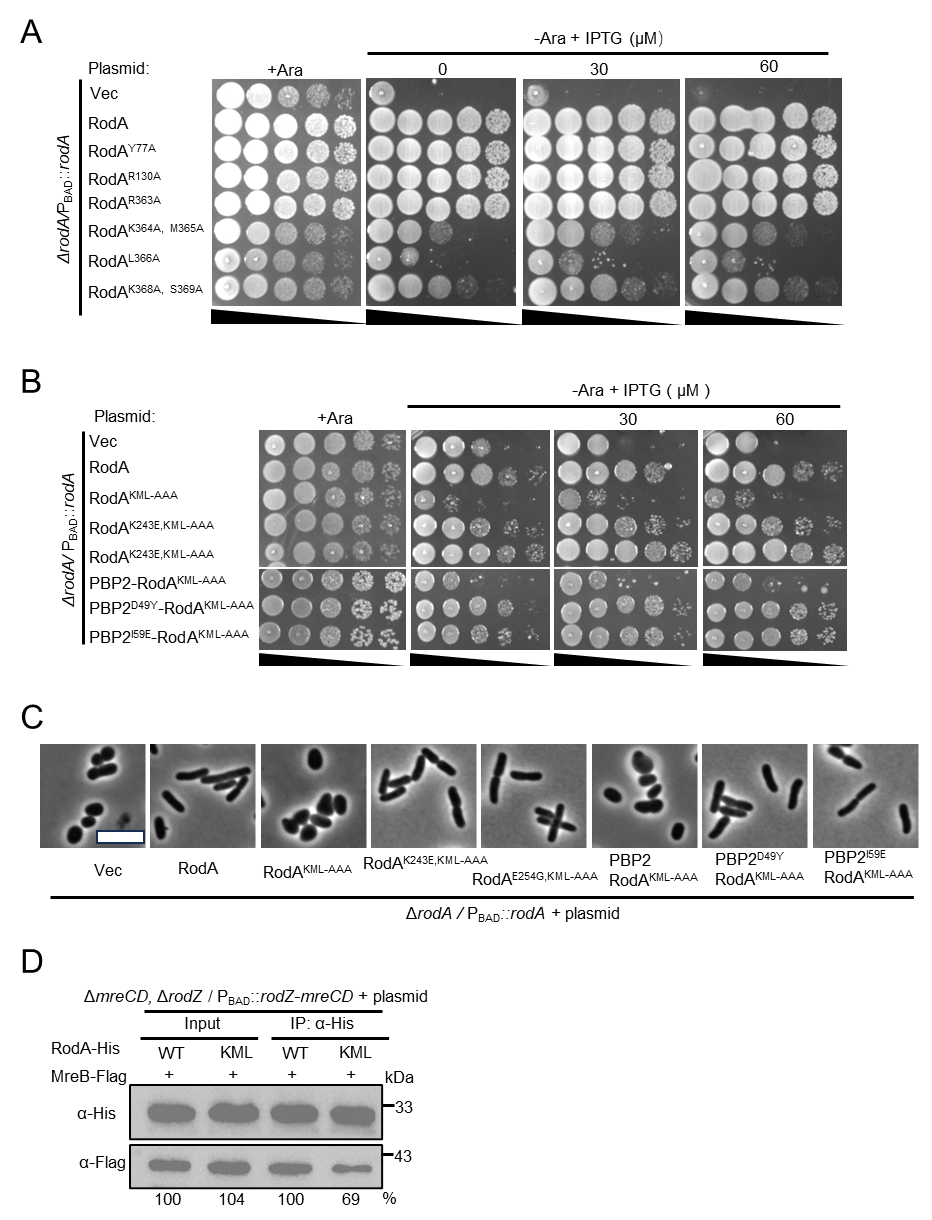


**Fig. S8 The C-terminal tail of RodA is important for its function in wild type cells but not in the presence of RodA-PBP2 active variants.** (A) RodA mutants failed to complement the RodA depletion strain. Plasmid harboring the indicated alleles of *rodA* were transformed into SD432 and transformants were selected on LB plates with ampicillin and IPTG. The spot test was performed as in Fig. 1A. (B) Activating mutations of RodA or PBP2 could suppress the growth defects caused by RodA^KML-AAA^. Plasmid pDSW210, pZR161, pSD314 or their derivatives were transformed into strain SD432 on LB plates with ampicillin, chloramphenicol and 0.2% arabinose at 37°C. The complementation test was performed as in Fig. 1A. (C) Activating RodA or PBP2 variants suppressed the shape defects caused by RodA^KML-AAA^. Overnight cultures of the strains from panel (B) were cultured, grown and immobilized on 2% agarose pads for photography as described in Fig. 1B. Scale bar, 5 μm. (D) RodA^KML-AAA^ displayed reduced interaction with MreB in RodZ-MreCD depleted cells as detected by Co-IP. Cells from overnight cultures of RZ96A carrying pZR318 or pZR318-KML were collected by centrifugation and washed twice with fresh LB medium, and then resuspended in the same volume of LB medium. The details of SDS-PAGE sample preparation are described in Methods. Intensitiy of each band was quantitated by Image J.


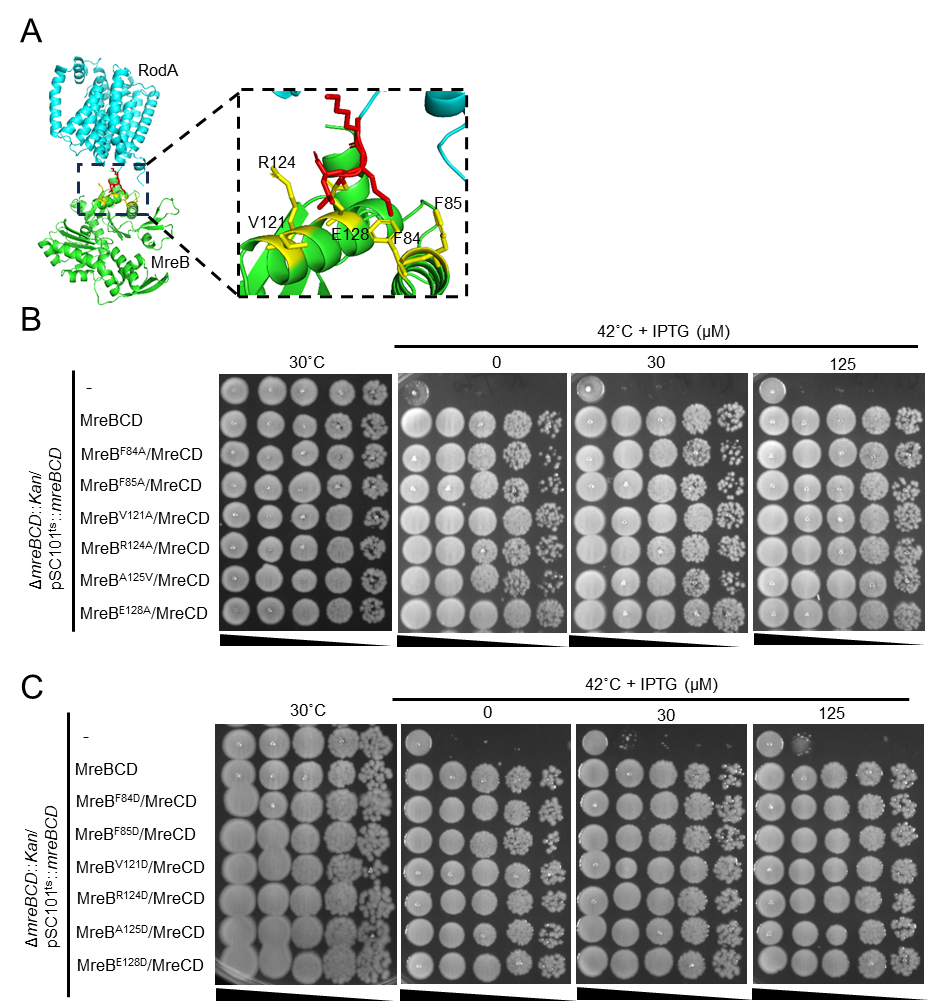


**Fig. S9 Characterization of residues on MreB that are potentially involved in its interaction with RodA.** (A) A structural model of the *E. coli* MreB-RodA complex obtained with AlphaFold3. The model was extracted from a structural model of the complete elongasome complex generated by AF3 (Fig. S4). Residues on MreB predicted to be involved in its interaction with RodA were shown in stick, colored red. (B-C) Complementation test of MreB mutants. Plasmid pDSW210, pSD316 or its derivatives with different *mreB* alleles were transformed into strain RZ49 and transformants were selected on LB plates with antibiotics and arabinose at 30°C.The spot test was performed as in Fig. 1A. Plates were incubated at 30°C (as a control) or 42°C overnight and photographed.

**Supplementary Text**

**Strain Construction**

**SD432**

The strain SD432 (W3110, *rodA<>aph* / pSD308 (P_BAD_::*rodA*)) was constructed by P1 transduction(2) of the *mrdB*<>aph cassette from P3962 (FB22(λFB185) [TB28, *mrdB<>aph* (*attλ bla lacI^q^* P_lac_::*mrdB*)] ) into strain W3110 / pSD308. Transductants were selected on LB plates with 25 μg/mL kanamycin, 15 μg/mL chloramphenicol, 8 mM sodium citrate and 0.2% arabinose at 37°C. Deletion of *mrdB* was verified by arabinose-dependent growth and loss of rod-shape upon the removal of arabinose.

**RZ49**

The strain RZ49 (TB28, *mreBCD<>aph* /pZR99 (pSC101^ts^::*mreBCD, addA^R^*)) was constructed by P1 transduction of the *mreBCD<>aph* cassette from P4206 ([TB28, MreBCD<>aph / pBAD33, P_ara_::*meBCD-le* *cat araC*]) into strain TB28 / pZR99 (pSC101^ts^::*mreBCD, addA^R^*). Transductants were selected on LB plates with 25 μg/mL kanamycin, 8 mM sodium citrate and 0.2% arabinose at 30°C. Deletion of *mreBCD* was verified by temperature-dependent growth.

**RZ50**

The strain RZ50 (TB28, *mrdAB<>aph*/pSD312 (P_BAD_::*pbp2-rodA*)) was constructed by P1 transduction of the *mrdAB<>aph* cassette from P3988 ([TB28, *mrdAB<>aph* (*attλ bla lacI^q^* P_lac_::*mrdAB*)]) into strain TB28 / pSD312. Transductants were selected on LB plates with 25 μg/mL kanamycin, 15 μg/mL chloramphenicol, 8 mM sodium citrate and 0.2% arabinose at 37°C. Deletion of *mrdAB* was verified by arabinose-dependent growth and loss of rod-shape upon the removal of arabinose.

**SD458-D49Y**

Strain SD458-D49Y were obtained by replacing the chromosomal *pbp2* allele of SD458 (TB28, ∆*rlpA::cat*) with respective *pbp^D49Y^* alleles from plasmid pZR109 (pSC101^ts^, *pbp2-rodA**)(2). Presence of the desired allele in each strain was verified by amplification and sequencing of the chromosomal DNA using primers 5-PBP2-SeqF and 3-RodA-seqR. Strains SD458-L61R, SD458-K243E and SD458-E254G were constructed similarly. Detailed procedures for the construction of these strains were provided in the Method section.

**RZ56-RZ61**

Strains carrying the *rodZ<>aph* cassette were obtained by P1-mediated transduction of *rodZ<>aph* cassette from P5439 ([TB28, *rodZ<>aph* / *ColE1, bla lacI^q^* P_lac_::*rodZ*]) into indicated strains. Transductants were selected on LB plates containing 25 μg/mL kanamycin, 15 μg/mL chloramphenicol and 8 mM sodium citrate at 37°C. Deletion of *rodZ* was verified by the loss of rod-shape of cells.

**RZ95 and RZ95A**

The strain RZ95 (TB28, *rodZ<>aph*/pZR186 (P_BAD_::*rodZ-mreCD*)) was constructed by P1 transduction of the *rodZ<>aph* cassette from P5439 (FB60/pFB290 [TB28, *rodZ<>aph* / *ColE1, bla lacI^q^* P_lac_::*rodZ*]) into strain TB28 / pZR186. Transductants were selected on LB plates with 25 μg/mL kanamycin, 15 μg/mL chloramphenicol, 8 mM sodium citrate and 0.2% arabinose at 37°C.

The strain RZ95A (TB28, *rodZ<>frt* / pZR186 (P_BAD_::*rodZ-mreCD*)) was constructed by transforming with pCP20 to eliminate antibiotic resistance gene of strain RZ95. Ampicillin-resistant transformants were selected at 30°C, purified once at 42°C and then tested for the loss of kanamycin resistance. Deletion of *rodZ* was verified by arabinose-dependent growth and loss of rod-shape upon the removal of arabinose.

**RZ96 and RZ96A**

The strain RZ96 (TB28, *rodZ<>frt*, *mreCD<>aph /* pZR186 (P_BAD_::*rodZ-mreCD*)) was constructed by P1 transduction of the *mreCD<>aph* cassette from SD464 (TB28, *mreCD<>aph*/ pSD352 (P_BAD_::*mreCD*)) into strain RZ95A. Transductants were selected on LB plates with 25 μg/mL kanamycin, 15 μg/mL chloramphenicol, 8 mM sodium citrate and 0.2% arabinose at 37°C. Transductants should be resistant to kanamycin and chloramphenicol, and depend on arabinose for growth.

The strain RZ96A (TB28, *rodZ<>frt*, *mreCD<>frt/* pZR186 (P_BAD_::*rodZ-mreCD*)) was constructed by transforming with pCP20 to eliminate the antibiotic resistance gene of strain RZ96. Ampicillin-resistant transformants were selected at 30°C, purified once at 42°C and then tested for the loss of kanamycin resistance. The correct strain should be resistant to chloramphenicol, and depend on arabinose for growth.

**Plasmids Construction**

The plasmid pSD308 (pBAD33, P_BAD_::*rodA*, *Cam^R^*) was constructed by ligation of an XbaI /HindIII digested DNA fragment containing *rodA* into pBAD33 digested with the same enzymes. The DNA fragment was amplified from W3110 chromosomal DNA using primers 5-XbaI-RodA and HindIII-RodA-3.

The plasmid pSD312 (pBAD33, P_BAD_::*pbp2-rodA*, *Cam^R^*) was constructed by ligation of an XbaI/HindIII digested DNA fragment containing *pbp2-rodA* into pBAD33 digested with the same enzymes. The DNA fragment was amplified from W3110 chromosomal DNA using primers 5-XbaI-PBP2 and HindIII-mrdB-3.

The plasmid pSD314 (pDSW210, *bla* P_206_::*pbp2-rodA*) was constructed by ligation of an EcoRI/HindIII digested DNA fragment containing *pbp2-rodA* into pDSW210 digested with the same enzymes. The DNA fragment was amplified from W3110 chromosomal DNA using primers 5-EcoRI-MrdA and 3-MrdB-HindIII. Derivatives of pSD314 carrying different alleles of *pbp2-rodA* were obtained by site-directed mutagenesis using primer pairs listed in Supplementary Table S3.

The plasmid pSD316 (pDSW210, *bla* P_206_::*mreBCD*) was constructed by ligation of an EcoRI/HindIII digested DNA fragment containing *mreBCD* into pDSW210 digested with the same enzymes. The DNA fragment was amplified from W3110 chromosomal DNA using primers 5-EcoRI-MreB-U and 3-MreD-HindIII-U. Derivatives of pSD316 carrying different alleles of *mreB* were obtained by site-directed mutagenesis using primer pairs listed in Supplementary Table S3.

The plasmid pZR11 (pMB1, *bla* P_lac_::*t18-pbp2)* was constructed by ligation of an XbaI/EcoRI digested DNA fragment containing *pbp2* into pUT18C digested with the same enzymes. The DNA fragment was amplified from W3110 chromosomal DNA using primers T18C-XbaI-PBP2-U and PBP2-EcoRI-T18C-U. Derivatives of pZR11 carrying different alleles of *pbp2* were obtained by site-directed mutagenesis using primer pairs listed in Table S3.

The plasmid pZR12 (pACYC184, *kan* P_lac_::*t25-pbp2*) was constructed by ligation of an XbaI/EcoRI digested DNA fragment containing *pbp2* into pKT25 digested with the same enzymes. The DNA fragment was amplified from W3110 chromosomal DNA using primers T25-XbaI-PBP2 and PBP2-EcoRI-T25C. Derivatives of pZR12 carrying different alleles of *pbp2* were obtained by site-directed mutagenesis using primer pairs listed in Table S3.

The plasmid pZR13 (pMB1, *bla* P_lac_::*t18-rodA)* was constructed by ligation of an XbaI/KpnI digested DNA fragment containing *rodA* into pUT18C digested with the same enzymes. The DNA fragment was amplified from W3110 chromosomal DNA using primers T18C-XbaI-RodA-U and RodA-KpnI-T18C-U. Derivatives of pZR13 carrying different alleles of *rodA* were obtained by site-directed mutagenesis using primer pairs listed in Table S3.

The plasmid pZR14 (pACYC184, *kan* P_lac_::*t25-rodA*) was constructed by ligation of an XbaI/KpnI digested DNA fragment containing *rodA* into pKT25 digested with the same enzymes. The DNA fragment was amplified from W3110 chromosomal DNA using primers T25C-XbaI-RodA-U and RodA-KpnI-T25C-U. Derivatives of pZR14 carrying different alleles of *rodA* were obtained by site-directed mutagenesis using primer pairs listed in Table S3.

The plasmid pZR25 (ColE1, P_lac_::*mreB’-t18C-mreB’*, Amp^R^) was constructed by ligation of an XhoI/AscI digested DNA fragment containing *t18c* fragment into pHC892 (*bla lacI^q^* P_lac_::*mreB’-mNeonGreen-‘mreB*) digested with the same enzymes. The DNA fragment was amplified from pUT18C using primers 5-XhoI-t25 and 3-T25-AscI. The *mNeonGreen cassette inserted in the middle of mreB* *(replacing the codon for G228).*

The plasmid pZR26 (ColE1, P_lac_::*mreB’-t25C-mreB’*, Amp^R^) was constructed by ligation of an XhoI/AscI digested DNA fragment containing *t25c* fragment into pHC892 digested with the same enzymes. The DNA fragment was amplified from pKT25 using primers 5-XhoI-t25 and 3-T25-AscI.

The plasmid pZR27 (pMB1, bla P_lac_::*mreB’-t18C-mreB’*, Amp^R^) was constructed by ligation of an XbaI/EcoRI digested DNA fragment containing mreB’-t18C-mreB’ fragment into pUT18C digested with the same enzymes. The DNA fragment was amplified from pZR25 using primers T18C-XbaI-mreB and T18C-EcoRI-MreB. Derivatives of pZR27 carrying different alleles of *mreB* were obtained by site-directed mutagenesis using primer pairs listed in Table S3.

The plasmid pZR28 (pACYC184, Kan P_lac_::*mreB’-t25c-mreB’*) was constructed by ligation of an XbaI/EcoRI digested DNA fragment containing t25C-mreB’-t25c fragment into pKT25 digested with the same enzymes. The DNA fragment was amplified from pZR26 using primers T18C-XbaI-mreB and T18C-EcoRI-MreB.

The plasmid pZR99 (pSC101^ts^::*mreBCD, addA^R^*) was constructed by ligation of an HindIII/ EcoRI digested DNA fragment containing *pbp2-rodA* into pHGB2 digested with the same enzymes. The DNA fragment was amplified from plasmid pSD314 using primers 5-MreB-HindIII and 3-mreD-EcoRI.

The plasmid pZR109 (pSC101^ts^::*pbp2-rodA, addA^R^*) was constructed by ligation of an HindIII/ EcoRI digested DNA fragment containing *pbp2-rodA* into pHGB2 digested with the same enzymes. The DNA fragment was amplified from plasmid pSD314 using primers 5-HindIII-PBP2 and 3-RodA-EcoRI. Derivatives of pZR109 carrying different alleles of *pbp2 or rodA* were obtained by site-directed mutagenesis using primer pairs listed in Table S3.

The plasmid pZR161 (pDSW210, *bla* P_206_::*rodA*) was constructed by ligation of an EcoRI/HindIII digested DNA fragment containing *rodA* into pDSW210 digested with the same enzymes. The DNA fragment was amplified from plasmid pSD314 using primers 5-EcoRI-RodA-U and 3-RodA-HindIII-U. Derivatives of pZR161 carrying different alleles of *rodA* were obtained by site-directed mutagenesis using primer pairs listed in Table S3.

The plasmid pZR180 (pDSW209, *bla* P_206_::*gfp-pbp2-rodA*) was constructed by ligation of an EcoRI/HindIII digested DNA fragment containing *gfp-pbp2-rodA* into pDSW209 digested with the same enzymes. The DNA fragment was amplified from W3110 chromosomal DNA using primers 5-EcoRI-gfp-PBP2 and HindIII-RodA-3. Derivatives of pZR180 carrying different alleles of *gfp-pbp2-rodA* were obtained by site-directed mutagenesis using primer pairs listed in Supplementary Table S3.

The plasmid pZR186 (pBAD33, P_BAD_::*rodZ-mreCD*, *Cam^R^*) was constructed by ligation of an KpnI/XbaI digested DNA fragment containing *rodZ* into pSD352 digested with the same enzymes. The DNA fragment was amplified from W3110 chromosomal DNA using primers 5-KpnI-RodZ and 3-XbaI-RodZ.

The plasmid pZR187 (pDSW210, *bla* P_206_::*rodZ-mreCD*) was constructed by ligation of an KpnI/HindIII digested DNA fragment containing *rodZ-mreCD* into pDSW210 digested with the same enzymes. The DNA fragment was amplified from plasmid pZR186 using primers 5-KpnI-RodZ and 3-mreD-HindIII.

The plasmid pZR288 (pDSW210, *bla* P_206_::*mreB-6xhis*) was constructed by ligation of an XbaI/HindIII digested DNA fragment containing *mreB-6xhis* into pDSW210 digested with the same enzymes. The DNA fragment was amplified from plasmid W3110 using primers 5-XbaI-MreB and 3-MreB-His-HindIII.

The plasmid pZR289 (pDSW210, *bla* P_206_::*gfp-pbp2/mreB-6xhis*) was constructed by ligation of an EcoRI/HindIII digested DNA fragment containing *gfp-pbp2/mreB-6xhis* into pDSW210 digested with the same enzymes. The *gfp-pbp2/mreB-6xhis* DNA fragment was obtained by overlap extension PCR. Two DNA fragments were amplified from pZR180 and pZR288 using primer pairs 5-EcoRI-GFP-U/3-MreB-XbaI-PBP2 and 5-PBP2-XbaI-MreB / 3-MreB-His-HindIII.

The plasmid pZR313 (pDSW210, *bla* P_206_::*rodA-6xhis*) was constructed by ligation of an XbaI/HindIII digested DNA fragment containing *rodA-6xhis* into pDSW210 digested with the same enzymes. The DNA fragment was amplified from plasmid W3110 using primers 5-XbaI-RodA-U and 3-RodA-His-HindIII-U. Derivatives of pZR313 carrying different alleles of *rodA* were obtained by site-directed mutagenesis using primer pairs listed in Table S3.

The plasmid pZR314 (pDSW210, *bla* P_206_::*mreB-flag*) was constructed by ligation of an XbaI/HindIII digested DNA fragment containing *mreB-flag* into pDSW210 digested with the same enzymes. The DNA fragment was amplified from plasmid W3110 using primers 5-XbaI-MreB-U and 3-Flag-L-MreB-HindIII. Derivatives of pZR314 carrying different alleles of *mreB* were obtained by site-directed mutagenesis using primer pairs listed in Table S3.

The plasmid pZR318 (pDSW210, *bla* P_206_::*mreB-flag/rodA-6xhis*) was constructed by ligation of an XbaI/HindIII digested DNA fragment containing *mreB-flag/rodA-6xhis* into pDSW210 digested with the same enzymes. The *mreB-flag/rodA-6xhis* DNA fragment was obtained by overlap extension PCR. Two DNA fragments were amplified from pZR313 and pZR314 using primer pairs 5-XbaI-MreB-U/3-RodA-Flag and 5-Flag-RodA/3-HindIII-6His-RodA-U. Derivatives of pZR318 carrying different alleles of *rodA* were obtained by site-directed mutagenesis using primer pairs listed in Table S3.

**S1 Table. Bacterial strains used in this study**

| Strain | Genotype | Source |
| --- | --- | --- |
| BTH101 | *cya-99 araD139 galE15 galK16 rpsL1 hsdR2 mcrA1 mcrB1* | (3) |
| JS238 | MC1061*, malPp*::*lacIQ srlC*::*Tn10 recA1* | (4) |
| TB28 | MG1655, Δ*lacIZYA*::*frt* | (5) |
| P4206 | FB30/pFB174 [TB28, *mreBCD<>aph* / pBAD33, P_BAD_::*mreBCD-le cat* araC] | (6) |
| P3962 | FB22(λFB185) [TB28, *mrdB<>aph* (*attλ bla lacI^q^* P_lac_::*mrdB*)]) | (6, 7) |
| SD432 | W3110, *rodA*::*aph* / pSD308(P_BAD_::*rodA*) | This study |
| SD458 | TB28, Δ*rlpA*::*cat* | This study |
| SD458-D49Y | TB28, Δ*rlpA*::*cat*, *pbp2^D49Y^* | This study |
| SD458-L61R | TB28, Δ*rlpA*::*cat*, *pbp2^L61R^* | This study |
| SD458-K243E | TB28, Δ*rlpA*::*cat*, *rodA^K243E^* | This study |
| SD458-E254G | TB28, Δ*rlpA*::*cat*, *rodA^E254G^* | This study |
| RZ49 | TB28, *mreBCD*::*aph*/pZR99(pSC101^ts^::*mreBCD*) | This study |
| RZ50 | TB28, *mrdAB*::*aph*/pSD312(P_BAD_::*pbp2-rodA*) | This study |
| RZ56 | TB28, Δ*rlpA*::*ca*t, *rodZ::kan* | This study |
| RZ57 | TB28, Δ*rlpA*::*cat* *pbp2^D49Y^*, Δ*rodZ*::*kan* | This study |
| RZ58 | TB28, Δ*rlpA*::*cat* *pbp2^L61R^*, Δ*rodZ*::*kan* | This study |
| RZ60 | TB28, Δ*rlpA*::*cat* *rodA^K243E^*, Δ*rodZ*::*kan* | This study |
| RZ61 | TB28, Δ*rlpA*::*cat* *rodA^E254G^*, Δ*rodZ*::*kan* | This study |
| RZ95 | TB28, *rodZ<>aph*/pZR186 (P_BAD_::*rodZ-mreCD*) | This study |
| RZ95A | TB28, *rodZ<>frt*/pZR186 (P_BAD_::*rodZ-mreCD*) | This study |
| RZ96 | TB28, *rodZ<>frt*, *mreCD*::*aph* / pZR186 (P_BAD_::*rodZ-mreCD*) | This study |
| RZ96A | TB28, *rodZ<>frt,* *mreCD*::*frt* / pZR186 (P_BAD_::*rodZ-mreCD*) | This study |

**S2 Table. Plasmids used in this study**

| Plasmid | Genotype | Source |
| --- | --- | --- |
| pDSW209 | pDSW206-*gfp-MCS*, *Amp^R^* | (8) |
| pDSW210 | pDSW206-*MCS-gfp, Amp^R^* | (8) |
| pKT25 | pACYC184, *kan* P_lac_::*t25-* | (3) |
| pUT18C | pMB1, *bla* P_lac_::*t18-* | (3) |
| pHC892 | *bla lacI^q^* P_lac_::*mreB’-mNeonGreen-‘mreB* | (9) |
| pHGB2 | pSC101^ts^, *Spc^R^* | (10) |
| pCP20 | pSC101^ts^, *cI857 repA^ts^, flp recombinase, Amp^R^* | (11) |
| pBAD33 | P_BAD_::*mcs, Cam^R^* | (12) |
| pSD308 | pBAD33, P_BAD_::*rodA*, *Cam^R^* | This study |
| pSD312 | pBAD33, P_BAD_::*pbp2-rodA*, *Cam^R^* | This study |
| pSD314 | pDSW210, P_206_::*pbp2-rodA*, *Amp^R^* | This study |
| pSD314-D49Y | pDSW210, P_206_::*pbp2^D49Y^-rodA*, *Amp^R^* | This study |
| pSD314-Y50E | pDSW210, P_206_::*pbp2^Y50E^-rodA*, *Amp^R^* | This study |
| pSD314-S54E | pDSW210, P_206_::*pbp2^S54E^-rodA*, *Amp^R^* | This study |
| pSD314-N57E | pDSW210, P_206_::*pbp2^N57E^-rodA*, *Amp^R^* | This study |
| pSD314-R58E | pDSW210, P_206_::*pbp2^R58E^-rodA*, *Amp^R^* | This study |
| pSD314-I59E | pDSW210, P_206_::*pbp2^I59E^-rodA*, *Amp^R^* | This study |
| pSD314-K60E | pDSW210, P_206_::*pbp2^K60E^-rodA*, *Amp^R^* | This study |
| pSD314-K243E | pDSW210, P_206_::*pbp2-rodA^K243E^*, *Amp^R^* | This study |
| pSD314-E254G | pDSW210, P_206_::*pbp2-rodA^E254G^*, *Amp^R^* | This study |
| pSD314-MalF16 | pDSW210, P_206_::*^malF16^pbp2-rodA*, *Amp^R^* | This study |
| pSD314-MalF16-D49Y | pDSW210, P_206_::*^malF16^pbp2^D49Y^-rodA*, *Amp^R^* | This study |
| pSD314-MalF16-I59E | pDSW210, P_206_::*^malF16^pbp2^I59E^-rodA*, *Amp^R^* | This study |
| pSD314-MalF16-K243E | pDSW210, P_206_::*^malF16^pbp2-rodA^K243E^*, *Amp^R^* | This study |
| pSD314-MalF16-E254G | pDSW210, P_206_::*^malF16^pbp2-rodA^E254G^*, *Amp^R^* | This study |
| pSD314-KML-AAA | pDSW210, P_206_::*pbp2-rodA^KML-AAA^*, *Amp^R^* | This study |
| pSD314-D49Y-KML | pDSW210, P_206_::*pbp2^D49Y^-rodA^KML-AAA^*, *Amp^R^* | This study |
| pSD314-I59E-KML | pDSW210, P_206_::*pbp2^I59E^-rodA^KML-AAA^*, *Amp^R^* | This study |
| pSD314-K243E-KML | pDSW210, P_206_::*pbp2-rodA^K243E,KML-AAA^*, *Amp^R^* | This study |
| pSD314-E254G-KML | pDSW210, P_206_::*pbp2-rodA^E254G,KML-AAA^*, *Amp^R^* | This study |
| pSD314-MalF16-KML | pDSW210, P_206_::*^malF16^pbp2-rodA^KML-AAA^*, *Amp^R^* | This study |
| pSD314-MalF16-D49Y-K243E-KML | pDSW210, P_206_::*^malF16^pbp2^D49Y^-rodA^K243E,KML-AAA^*, *Amp^R^* | This study |
| pSD314-MalF16-I59E-K243E-KML | pDSW210, P_206_::*^malF16^pbp2^I59E^-rodA^K243E,KML-AAA^*, *Amp^R^* | This study |
| pSD316 | pDSW210, P_206_::*mreBCD*, *Amp^R^* | This study |
| pSD316-S51A | pDSW210, P_206_::*mreB^S51A^/mreCD*, *Amp^R^* | This study |
| pSD316-V52A | pDSW210, P_206_::*mreB^V52A^/mreCD*, *Amp^R^* | This study |
| pSD316-F84A | pDSW210, P_206_::*mreB^F84A^/mreCD*, *Amp^R^* | This study |
| pSD316-F85A | pDSW210, P_206_::*mreB^F85A^/mreCD*, *Amp^R^* | This study |
| pSD316-V86A | pDSW210, P_206_::*mreB^V86A^/mreCD*, *Amp^R^* | This study |
| pSD316-K89A | pDSW210, P_206_::*mreB^K89A^/mreCD*, *Amp^R^* | This study |
| pSD316-H93A | pDSW210, P_206_::*mreB^H93A^/mreCD*, *Amp^R^* | This study |
| pSD316-K96A | pDSW210, P_206_::*mreB^K96A^/mreCD*, *Amp^R^* | This study |
| pSD316-V121A | pDSW210, P_206_::*mreB^V121A^/mreCD*, *Amp^R^* | This study |
| pSD316-R124A | pDSW210, P_206_::*mreB^R124A^/mreCD*, *Amp^R^* | This study |
| pSD316-A125V | pDSW210, P_206_::*mreB^A125V^/mreCD*, *Amp^R^* | This study |
| pSD316-E128A | pDSW210, P_206_::*mreB^E128A^/mreCD*, *Amp^R^* | This study |
| pSD316-F84D | pDSW210, P_206_::*mreB^F84D^/mreCD*, *Amp^R^* | This study |
| pSD316-F85D | pDSW210, P_206_::*mreB^F85D^/mreCD*, *Amp^R^* | This study |
| pSD316-V121D | pDSW210, P_206_::*mreB^V121D^/mreCD*, *Amp^R^* | This study |
| pSD316-R124D | pDSW210, P_206_::*mreB^R124D^/mreCD*, *Amp^R^* | This study |
| pSD316-A125D | pDSW210, P_206_::*mreB^A125D^/mreCD*, *Amp^R^* | This study |
| pSD316-E128D | pDSW210, P_206_::*mreB^E128D^/mreCD*, *Amp^R^* | This study |
| pZR25 | ColE1, P_lac_::*mreB’-t18C-mreB’*, Amp^R^ | This study |
| pZR26 | ColE1, P_lac_::*mreB’-t25C-mreB’*, Amp^R^ | This study |
| pZR27 | pMB1, *bla* P_lac_::*mreB’-t18C-mreB’* | This study |
| pZR27-K89A | pMB1, *bla* P_lac_::*mreB’-t18C-mreB’-K89A* | This study |
| pZR28 | pACYC184, *kan* P_lac_::*mreB’-t25C-mreB’* | This study |
| pZR11 | pMB1, *bla* P_lac_::*t18-pbp2* | This study |
| pZR12 | pACYC184, *kan* P_lac_::*t25-pbp2* | This study |
| pZR12-MalF16 | pACYC184, *kan* P_lac_::*t25-^malF16^pbp2* | This study |
| pZR13 | pMB1, *bla* P_lac_::*t18-rodA* | This study |
| pZR13-KML-AAA | pMB1, *bla* P_lac_::*t18-rodA^KML-AAA^* | This study |
| pZR14 | pACYC184, *kan* P_lac_::*t25-rodA* | This study |
| pZR99 | pSC101^ts^::*mreBCD, addA^R^* | This study |
| pZR109 | pSC101^ts^::*pbp2-rodA, addA^R^* | This study |
| pZR109-D49Y | pSC101^ts^::*pbp2^D49Y^-rodA, addA^R^* | This study |
| pZR109-L61R | pSC101^ts^::*pbp2^L61R^-rodA, addA^R^* | This study |
| pZR109-K243E | pSC101^ts^::*pbp2-rodA^K243E^, addA^R^* | This study |
| pZR109-E254G | pSC101^ts^::*pbp2-rodA^E254G^, addA^R^* | This study |
| pZR161 | pDSW210, *bla* P_206_::*rodA* | This study |
| pZR161-Y226A | pDSW210, *bla* P_206_::*rodA^Y226A^* | This study |
| pZR161-Y226E | pDSW210, *bla* P_206_::*rodA^Y226E^* | This study |
| pZR161-H227D | pDSW210, *bla* P_206_::*rodA^H227D^* | This study |
| pZR161-Q230A | pDSW210, *bla* P_206_::*rodA^Q230A^* | This study |
| pZR161-L253D | pDSW210, *bla* P_206_::*rodA^L253D^* | This study |
| pZR161-L253A | pDSW210, *bla* P_206_::*rodA^L253A^* | This study |
| pZR161-Q250E | pDSW210, *bla* P_206_::*rodA^Q250E^* | This study |
| pZR161-A234D | pDSW210, *bla* P_206_::*rodA^A234D^* | This study |
| pZR161-A234G | pDSW210, *bla* P_206_::*rodA^A234G^* | This study |
| pZR161-L240R | pDSW210, *bla* P_206_::*rodA^L240R^* | This study |
| pZR161-K243E | pDSW210, *bla* P_206_::*rodA^K243E^* | This study |
| pZR161-K243A | pDSW210, *bla* P_206_::*rodA^K243A^* | This study |
| pZR161-E254G | pDSW210, *bla* P_206_::*rodA^E254G^* | This study |
| pZR161-E254K | pDSW210, *bla* P_206_::*rodA^E254K^* | This study |
| pZR161-KM-AA | pDSW210, *bla* P_206_::*rodA^K364A,M365A^* | This study |
| pZR161-LS-AA | pDSW210, *bla* P_206_::*rodA^L366A,S367A^* | This study |
| pZR161-Y77A | pDSW210, *bla* P_206_::*rodA^Y77A^* | This study |
| pZR161-R130A | pDSW210, *bla* P_206_::*rodA^R130A^* | This study |
| pZR161-R363A | pDSW210, *bla* P_206_::*rodA^R363A^* | This study |
| pZR161-K364A | pDSW210, *bla* P_206_::*rodA^K364A^* | This study |
| pZR161-M365A | pDSW210, *bla* P_206_::*rodA^M365A^* | This study |
| pZR161-L366A | pDSW210, *bla* P_206_::*rodA^L366A^* | This study |
| pZR161-K368A | pDSW210, *bla* P_206_::*rodA^K368A^* | This study |
| pZR161-S369A | pDSW210, *bla* P_206_::*rodA^S369A^* | This study |
| pZR161-KML-AAA | pDSW210, *bla* P_206_::*rodA^K364A,M365A,L366A^* | This study |
| pZR180 | pDSW209, *bla* P_206_::*gfp-pbp2-rodA* | This study |
| pZR186 | pBAD33, P_BAD_::*rodZ-mreCD*, *Cam^R^* | This study |
| pZR187 | pDSW210, *bla* P_206_::*rodZ-mreCD* | This study |
| pZR219 | pBAD33, P_BAD_::*gfp-pbp2* | This study |
| pZR288 | pDSW210, *bla* P_206_::*mreB-6xhis* | This study |
| pZR289 | pDSW210, *bla* P_206_::*gfp-pbp2*-*mreB-6xhis* | This study |
| pZR289-MalF16 | pDSW210, *bla* P_206_::*gfp-^malF16^pbp2*-*mreB-6xhis* | This study |
| pZR313 | pDSW210, *bla* P_206_::*rodA-6xhis* | This study |
| pZR313-KML-AAA | pDSW210, *bla* P_206_::*rodA^KML-AAA^-6xhis* | This study |
| pZR314 | pDSW210, *bla* P_206_::*mreB-flag* | This study |
| pZR314-K89A | pDSW210, *bla* P_206_::*mreB^K89A^-flag* | This study |
| pZR318 | pDSW210, *bla* P_206_::*mreB-flag-rodA-6xhis* | This study |
| pZR318-KML | pDSW210, *bla* P_206_::*mreB-flag-rodA^KML-AAA^-6xhis* | This study |

**S3 Table. Primers used in this study**

| Primer name | Sequence |
| --- | --- |
| 5-RodZ-seqF | TTGTCACGAGCACACAGACGGTCT |
| 3-RodZ-seqR | ACTGTACGGCGATGGGAGCACCAT |
| 5-MreC-SeqF | TCGGATGCAGGCAGGGGAAGTG |
| 3-MreD-seqR | TGCTGCGCGCTCTCCTGCGG |
| 5-PBP2-SeqF | GTCTGTACCGGGCGTGGAGCATCA |
| 3-RodA-SeqR | GATTTGCCGTCGCGTTCAGTGGTTCG |
| 5-EcoRI-RodA | gcGAATTCCAGCGGAGGACCATTAATC |
| 3-HindIII-RodA | CCCAAGCTTGCTTACGCATTGCGCACC |
| 5-RodA-Y226A | CCACTCGGCGCGGGCgcTCACATTATTCAGTCT |
| 3-RodA-Y226A | AGACTGAATAATGTGAgcGCCCGCGCCGAGTGG |
| 5-rodA-Y226E | CCACTCGGCGCGGGCgaaCACATTATTCAGTCT |
| 3-RodA-Y226E | AGACTGAATAATGTGttcGCCCGCGCCGAGTGG |
| 5-RodA-H227D | CTCGGCGCGGGCTATGACATTATTCAGTCTAA |
| 3-RodA-H227D | TTAGACTGAATAATGTCATAGCCCGCGCCGAG |
| 5-RodA-H227A | CTCGGCGCGGGCTATGCCATTATTCAGTCTAAA |
| 3-RodA-H227A | TTAGACTGAATAATGGCATAGCCCGCGCCGAG |
| 5-RodA-Q230A | GGCTATCACATTATTGCGTCTAAAATTGCTATT |
| 3-RodA-Q230A | AATAGCAATTTTAGACGCAATAATGTGATAGCC |
| 5-RodA-Q230E | GGCTATCACATTATTGAGTCTAAAATTGCTATT |
| 3-RodA-Q230E | AATAGCAATTTTAGACTCAATAATGTGATAGCC |
| 5-RodA-A234D | ATTCAGTCTAAAATTGATATTGGCTCCGGCGGA |
| 3-RodA-A234D | TCCGCCGGAGCCAATATCAATTTTAGACTGAAT |
| 5-RodA-A234G | ATTCAGTCTAAAATTGgTATTGGCTCCGGCGGA |
| 3-RodA-A234G | TCCGCCGGAGCCAATAcCAATTTTAGACTGAAT |
| 5-RodA-Q250A | TGGCTGCACGGCACTGCGTCACAGCTTGAATTT |
| 3-RodA-Q250A | AAATTCAAGCTGTGACGCAGTGCCGTGCAGCCA |
| 5-RodA-Q250E | TGGCTGCACGGCACTGAGTCACAGCTTGAATTT |
| 3-RodA-Q250E | AAATTCAAGCTGTGACTCAGTGCCGTGCAGCCA |
| 5-RodA-L253D | GGCACTCAGTCACAGGATGAATTTCTCCCCGAA |
| 3-RodA-L253D | TTCGGGGAGAAATTCATCCTGTGACTGAGTGCC |
| 5-RodA-L240R | ATTGGCTCCGGCGGAAGACGCGGCAAAGGCTGG |
| 3-RodA-L240R | CCAGCCTTTGCCGCGTCTTCCGCCGGAGCCAAT |
| 5-RodA-K243E | GGCGGATTACGCGGCGAAGGCTGGCTGCACGGC |
| 3-RodA-K243E | GCCGTGCAGCCAGCCTTCGCCGCGTAATCCGCC |
| 5-RodA-E254G | ACTCAGTCACAGCTTGGATTTCTCCCCGAACGC |
| 3-RodA-E254G | GCGTTCGGGGAGAAATCCAAGCTGTGACTGAGT |
| 5-rodA-E254K | ACTCAGTCACAGCTTaAATTTCTCCCCGAACGC |
| 3-rodA-E254K | GCGTTCGGGGAGAAATTtAAGCTGTGACTGAGT |
| 5-EcoRI-PBP2 | gcGaattcTTTGAGTAGAAAACGCAGCGG |
| 3-RodA-HindIII | CCCAAGCTTGCTTACGCATTGCGCACC |
| 5-PBP2-F47E | CTGCAAATTGTTCGCGAAACCGACTACCAGACC |
| 3-PBP2-F47E | GGTCTGGTAGTCGGTTTCGCGAACAATTTGCAG |
| 5-PBP2-T48E | CAAATTGTTCGCTTTGAAGACTACCAGACCCGC |
| 3-PBP2-T48E | GCGGGTCTGGTAGTCTTCAAAGCGAACAATTTG |
| 5-PBP2-D49Y | ATTGTTCGCTTTACCTACTACCAGACCCGCTCT |
| 3-PBP2-D49Y  5-PBP2-Y50E | AGAGCGGGTCTGGTAGTAGGTAAAGCGAACAAT  GTTCGCTTTACCGACGAACAGACCCGCTCTAAT |
| 3-PBP2-Y50E | ATTAGAGCGGGTCTGTTCGTCGGTAAAGCGAAC |
| 3-PBP2-R53E | GCGGTTTTCATTAGATTCGGTCTGGTAGTCGGT |
| 5-PBP2-R53E | ACCGACTACCAGACCGAATCTAATGAAAACCGC |
| 5-PBP2-S54E | GACTACCAGACCCGCGAAAATGAAAACCGCATT |
| 3-PBP2-S54E | AATGCGGTTTTCATTTTCGCGGGTCTGGTAGTC |
| 3-PBP2-N55E | CTTAATGCGGTTTTCTTCAGAGCGGGTCTGGTA |
| 5-PBP2-N55E | TACCAGACCCGCTCTGAAGAAAACCGCATTAAG |
| 5-PBP2-E56K | CAGACCCGCTCTAATAAAAACCGCATTAAGCTG |
| 3-PBP2-E56K | CAGCTTAATGCGGTTTTTATTAGAGCGGGTCTG |
| 5-PBP2-N57E | ACCCGCTCTAATGAAGAACGCATTAAGCTGGTG |
| 3-PBP2-N57E | CACCAGCTTAATGCGTTCTTCATTAGAGCGGGT |
| 5-PBP2-R58E | CGCTCTAATGAAAACGAAATTAAGCTGGTGCCT |
| 3-PBP2-R58E | AGGCACCAGCTTAATTTCGTTTTCATTAGAGCG |
| 5-PBP2-I59E | TCTAATGAAAACCGCGAAAAGCTGGTGCCTATC |
| 3-PBP2-I59E | GATAGGCACCAGCTTTTCGCGGTTTTCATTAGA |
| 5-PBP2-K60E | AATGAAAACCGCATTGAACTGGTGCCTATCGCG |
| 3-PBP2-K60E | CGCGATAGGCACCAGTTCAATGCGGTTTTCATT |
| 3-RodA-K364A | GCTTTTCGACAACATTGCCCTGTGGGTGTGGAT |
| 5-RodA-K364A | ATCCACACCCACAGGGCAATGTTGTCGAAAAGC |
| 5-RodA-K368A | AGGAAAATGTTGTCGAGCAGCGTGTAAGAGGTG |
| 3-RodA-K368A | CACCTCTTACACGCTGCTCGACAACATTTTCCT |
| 3-RodA-S369A | GCGCACCTCTTACACGGCTTTCGACAACATTTT |
| 5-RodA-S369A | AAAATGTTGTCGAAAGCCGTGTAAGAGGTGCGC |
| 5-RodA-V370A | ATGTTGTCGAAAAGCGCGTAAGAGGTGCGCAAT |
| 3-RodA-V370A | ATTGCGCACCTCTTACGCGCTTTTCGACAACAT |
| 3-RodA-L366A | TTACACGCTTTTCGACGCCATTTTCCTGTGGGT |
| 5-RodA-L366A | ACCCACAGGAAAATGGCGTCGAAAAGCGTGTAA |
| 5-RodA-KML-AAA | ATCCACACCCACAGGGCAGCGGCGTCGAAAAGCGTGTAAGA |
| 3-RodA-KML-AAA | TCTTACACGCTTTTCGACGCCGCTGCCCTGTGGGTGTGGAT |
| 5-MreB-PKSV-AAAA | GATCGTGCCGGTTCAGCGGCAGCCGCAGCTGCAGTAGGTCAT |
| 3-MreB-PKSV-AAAA | ATGACCTACTGCAGCTGCGGCTGCCGCTGAACCGGCACGATC |
| 5-EcoRI-MreB-U | CACACAGGAAACAGACCATGGAATTCGCTTTCAGGATTATCCCTTA |
| 5-HindIII-MreB-U | AATCTAGCGAGGGCTTTACTAAGCTTATGTTGAAAAAATTTCGTGG |
| 3-MreB-EcoRI-U | CTTACCCGTCTTACTGTCGGGAATTCTTACTCTTCGCTGAACAGGT |
| 5-XhoI-t25 | GACTCTCGAGCATGACCATGCAGCAATCGC |
| 3-T25-AscI | GATTGGCGCGCCTGCAGCCCGCCGCGTGC |
| 5-XhoI-t18 | GACTCTCGAGCGCCGCCAGCGAGGCCACGGG |
| 3-t18-AscI | GACTGGCGCGCCGCGTTCCACTGCGCCCAGCG |
| 5-KpnI-RodZ | ggGGTACCTCAATTCTCATTTAAACG |
| 3-mreD-HindIII | GACTAAGCTTGTCATAGAAACCTTTATTGCACTG |
| 5-EcoRI-MalF16-PBP2 | TGGAATTCATGGATGTCATTAAAAAGAAACATTGGTGGCAAAGCGACGCGCTGAAATTTGTGCGCCGGGCGCTG |
| HindIII-mrdB-3 | CCCAAGCTTGCTTACGCATTGCGCACC |
| T25-XbaI-PBP2 | GCTCTAGAGAAACTACAGAACTCTTTTCGC |
| PBP2-EcoRI-T25C | gcGAATTCTTAATGGTCCTCCGCTGCGGCA |
| pKT25-out-R | CCATTCGCCATTCAGGCTGCGCAACTGT |
| T25C-in-F | TTCGAGGCGGTCAAGGTGATCGGCAAT |
| 5-XbaI-PBP2-mal16 | GCTCTAGAGATGGATGTCATTAAAAAGA |
| T18C-XbaI-RodA | gcTCTAGAgACGGATAATCCGAATAAA |
| T18C-EcoRI-MreB | CGGAATTCTTACTCTTCGCTGAAC |
| T18C-XbaI-MreB | gcTCTAGAatgTTGAAAAAATTTCGTGGCAT |
| 5-pbad-XbaI-GFP-U | CTCGGTACCCGGGGATCCTCTAGAATGAGTAAAGGAGAAG |
| 3-HindIII-PBP2 | CCAAGCTTTTAATGGTCCTCCGCTGCGG |
| pBAD33-SeqR | GGCAAATTCTGTTTTATCAGACCG |
| pBAD33-SeqF | CTTTGCTATGCCATAGCATTTTTA |
| 5-XbaI-MreB | CCTCTAGAATGTTGAAAAAATTTCGTGG |
| 3-MreB-His-HindIII | CCAAGCTTTTAGTGATGGTGATGGTGATGCTCTTCGCTGAACAGGTCGCC |
| 5-EcoRI-GFP-U | CACACAGGAAACAGACCATGGAATTCATGAGTAAAGGAGAAGAACT |
| 5-PBP2-XbaI-MreB | CCGCAGCGGAGGACCATTAATCTAGAATGTTGAAAAAATTTCGTGG |
| 3-MreB-XbaI-PBP2 | CCACGAAATTTTTTCAACATTCTAGATTAATGGTCCTCCGCTGCGG |
| 3-HindIII-MreB-His-U | TCATCCGCCAAAACAGCCAAGCTTTTAGTGATGGTGATGGTGATGCTCTTCGCTGAACAGG |
| 5-XbaI-RodA-U | CTCGGTACCCGGGGATCCTCTAGAATGACGGATAATCCGAATAAAA |
| 3-RodA-Flag-U-HindIII | CGCCAAAACAGCCAAGCTTTTTACTTATCGTCGTCATCCTTGTAATCCACGCTTTTCGACAACATTT |
| 5-RodA-MreB-His | ATCACCATCACCATCACTAAATGACGGATAATCCGAATAA |
| 3-MreB-His-RodA | TTATTCGGATTATCCGTCATTTAGTGATGGTGATGGTGAT |
| 3-RodA-Flag-HindIII-U | CTCATCCGCCAAAACAGCCAAGCTTTTACTTATCGTCGTCATCCTTGTAATCCACGC |
| 5-XbaI-MreB-T18C-U | GAACGCCACTGCAGGTCGACTCTAGACTCACGAatgTTGAAAAAATTTCGTGGCAT |
| 3-EcoRI-MreB-T18C-U | TATTACTTAGTTATATCGATGAATTCTTACTCTTCGCTGAACAGGT |
| 3-MreD-HindIII-U | TCTCATCCGCCAAAACAGCCAAGCTTGTCATAGAAACCTTTATTGC |
| 5-EcoRI-MreB-U | CACACAGGAAACAGACCATGGAATTCGCTTTCAGGATTATCCCTTA |
| 5-MreB-S51A | GCCGGTTCACCGAAAGCCGTAGCTGCAGTAGGT |
| 3-MreB-S51A | ACCTACTGCAGCTACGGCTTTCGGTGAACCGGC |
| 5-MreB-V52A | GGTTCACCGAAAAGCGCAGCTGCAGTAGGTCAT |
| 3-MreB-V52A | ATGACCTACTGCAGCTGCGCTTTTCGGTGAACC |
| 5-MreB-F84D | GGCGTTATCGCCGACGACTTCGTGACTGAAAAA |
| 3-MreB-F84D | TTTTTCAGTCACGAAGTCGTCGGCGATAACGCC |
| 5-MreB-F85D | GTTATCGCCGACTTCGACGTGACTGAAAAAATG |
| 3-MreB-F85D | CATTTTTTCAGTCACGTCGAAGTCGGCGATAAC |
| 5-MreB-K89A | TTCTTCGTGACTGAAGCAATGCTCCAGCACTTC |
| 3-MreB-K89A | GAAGTGCTGGAGCATTGCTTCAGTCACGAAGAA |
| 5-MreB-V121D | GTTGGCGCGACCCAGGATGAACGCCGCGCAATT |
| 3-MreB-V121D | AATTGCGCGGCGTTCATCCTGGGTCGCGCCAAC |
| 5-MreB-R124D | ACCCAGGTTGAACGCGACGCAATTCGTGAATCC |
| 3-MreB-R124D | GGATTCACGAATTGCGTCGCGTTCAACCTGGGT |
| 5-MreB-A125D | CAGGTTGAACGCCGCGACATTCGTGAATCCGCG |
| 3-MreB-A125D | CGCGGATTCACGAATGTCGCGGCGTTCAACCTG |
| 5-MreB-E128D | CGCCGCGCAATTCGTGACTCCGCGCAGGGCGCT |
| 3-MreB-E128D | CAGCGCCCTGCGCGGAGTCACGAATTGCGCGGCGTT |
| 3-MreB-FLAG-HindIII-U | TCTCATCCGCCAAAACAGCCAAGCTTTTACTTATCGTCGTCATCCTTGT |

**References:**

1. Nygaard R, Graham CLB, Belcher Dufrisne M, Colburn JD, Pepe J, Hydorn MA, et al. Structural basis of peptidoglycan synthesis by E. coli RodA-PBP2 complex. Nat Commun. 2023;14(1).

2. Lennox ES. Transduction of linked genetic characters of the host by bacteriophage P1. Virology. 1955;1(2):190-206.

3. Karimova G, Dautin N, Ladant D. Interaction network among Escherichia coli membrane proteins involved in cell division as revealed by bacterial two-hybrid analysis. J Bacteriol. 2005;187(7):2233-43.

4. Pichoff S, Vollrath B, Touriol C, Bouché JP. Deletion analysis of gene minE which encodes the topological specificity factor of cell division in Escherichia coli. Mol Microbiol. 1995;18(2):321-9.

5. Bernhardt TG, de Boer PA. The Escherichia coli amidase AmiC is a periplasmic septal ring component exported via the twin-arginine transport pathway. Mol Microbiol. 2003;48(5):1171-82.

6. Bendezu FO, Hale CA, Bernhardt TG, de Boer PA. RodZ (YfgA) is required for proper assembly of the MreB actin cytoskeleton and cell shape in E. coli. EMBO J. 2009;28(3):193-204.

7. Bendezu FO, de Boer PA. Conditional lethality, division defects, membrane involution, and endocytosis in mre and mrd shape mutants of Escherichia coli. J Bacteriol. 2008;190(5):1792-811.

8. Weiss DS, Chen JC, Ghigo JM, Boyd D, Beckwith J. Localization of FtsI (PBP3) to the septal ring requires its membrane anchor, the Z ring, FtsA, FtsQ, and FtsL. J Bacteriol. 1999;181(2):508-20.

9. Cho H, Wivagg CN, Kapoor M, Barry Z, Rohs PDA, Suh H, et al. Bacterial cell wall biogenesis is mediated by SEDS and PBP polymerase families functioning semi-autonomously. Nat Microbiol. 2016;1:16172.

10. Churchward G, Belin D, Nagamine Y. A pSC101-derived plasmid which shows no sequence homology to other commonly used cloning vectors. Gene. 1984;31(1-3):165-71.

11. Datsenko KA, Wanner BL. One-step inactivation of chromosomal genes in Escherichia coli K-12 using PCR products. Proc Natl Acad Sci U S A. 2000;97(12):6640-5.

12. Guzman LM, Belin D, Carson MJ, Beckwith J. Tight regulation, modulation, and high-level expression by vectors containing the arabinose PBAD promoter. J Bacteriol. 1995;177(14):4121-30.
